# Anxiety, Depression and Quality of Life—A Systematic Review of Evidence from Longitudinal Observational Studies

**DOI:** 10.3390/ijerph182212022

**Published:** 2021-11-16

**Authors:** Johanna Katharina Hohls, Hans-Helmut König, Eleanor Quirke, André Hajek

**Affiliations:** Department of Health Economics and Health Services Research, University Medical Center Hamburg-Eppendorf, 20246 Hamburg, Germany; johannakatharinahohls@gmail.com (J.K.H.); h.koenig@uke.de (H.-H.K.); quirkeuke@outlook.com (E.Q.)

**Keywords:** anxiety, depression, quality of life, systematic review, meta-analysis, longitudinal study

## Abstract

This review aimed to systematically review observational studies investigating the longitudinal association between anxiety, depression and quality of life (QoL). A systematic search of five electronic databases (PubMed, PsycINFO, PSYNDEX, NHS EED and EconLit) as well as forward/backward reference searches were conducted to identify observational studies on the longitudinal association between anxiety, depression and QoL. Studies were synthesized narratively. Additionally, a random-effects meta-analysis was performed using studies applying the mental and physical summary scores (MCS, PCS) of the Short Form Health Survey. The review was prospectively registered with PROSPERO and a study protocol was published. *n* = 47 studies on heterogeneous research questions were included, with sample sizes ranging from *n* = 28 to 43,093. Narrative synthesis indicated that QoL was reduced before disorder onset, dropped further during the disorder and improved with remission. Before onset and after remission, QoL was lower in comparison to healthy comparisons. *n* = 8 studies were included in random-effects meta-analyses. The pooled estimates of QoL at follow-up (FU) were of small to large effect sizes and showed that QoL at FU differed by disorder status at baseline as well as by disorder course over time. Disorder course groups differed in their MCS scores at baseline. Effect sizes were generally larger for MCS relative to PCS. The results highlight the relevance of preventive measures and treatment. Future research should consider individual QoL domains, individual anxiety/depressive disorders as well as the course of both over time to allow more differentiated statements in a meta-analysis.

## 1. Introduction

The World Health Organization [1] estimates that 264 million people worldwide were suffering from an anxiety disorder and 322 million from a depressive disorder in 2015, corresponding to prevalence rates of 3.6% and 4.4%. While their prevalence varies slightly by age and gender [1], they are among the most common mental disorders in the general population [2,3,4,5,6]. During the COVID-19 pandemic, multiple challenges have arisen for many, such as loneliness [7] or financial hardship. A meta-analysis showed a prevalence of anxiety of about 32% (95% CI: 28–37) and a prevalence of depression (*n* = 14 studies) of about 34% (95% CI: 28–41) in general populations during the COVID-19 pandemic [8].

Anxiety and depression have been associated with adverse societal and individual correlates, including higher health care costs [9,10,11] and an increased risk for physical comorbidities, such as cardiovascular illnesses [12,13]. Moreover, they have been linked to a reduced quality of life (QoL) in numerous cross-sectional as well as longitudinal studies in which they significantly predicted QoL outcomes [14,15,16,17,18]. Other studies have reported a reverse association, whereby QoL was predictive of mental health outcomes [19] or a bi-directional association [20,21]. Some very recent studies also examined these associations among quite different samples (e.g., [22,23,24,25]).

Looking at longitudinal rather than cross-sectional data from observational studies has several advantages. It allows for the identification of trajectories over time within the same individuals rather than focusing on group differences at one point in time only [26]. Moreover, when appropriate methods are applied to longitudinal data, intraindividual heterogeneity can be taken into account, resulting in more consistent estimates [27]. This has previously been demonstrated in QoL research [28]. A need to analyze longitudinal changes in QoL domains in QoL research in people with mental disorders has also been previously identified [29]. Beyond individual longitudinal studies suggesting a link between anxiety or depression and QoL, several systematic reviews have synthesized longitudinal evidence on these associations and mostly reported negative associations between the variables. These reviews have tended to focus on specific age groups, such as older adults [30], samples with specific diseases [31,32], or have investigated the effect of specific treatments on QoL in patients with anxiety [33]. Investigating these associations in samples without these limitations could reduce the effect of specific conditions and treatments on the association and strengthen the conclusions that can be drawn.

In light of the previous findings, this study aims to add to the present literature by systematically synthesizing evidence from observational studies on the longitudinal association between anxiety, depression and QoL across all age groups in samples who do not have other specific illnesses and do not receive specific treatments.

## 2. Materials and Methods

This review was registered with PROSPERO (CRD42018108008) and a study protocol was published [34].

### 2.1. Search Strategy

Five electronic databases from several fields of research (PubMed, PsycINFO, PSYNDEX, NHS EED and EconLit) were examined until December 2020. Where possible, search terms were entered as Medical Subject Headings (MeSH) or as keywords in the title/abstract. The PubMed search strategy was: (anxi*[Title/Abstract] or depress*[Title/Abstract] or anxiety disorder[MeSH] or depressive disorder[MeSH]) and quality of life[MeSH] and longitudinal study[MeSH]. Please note that “*” is a truncation symbol. Time or location were not restricted. In addition, we applied backward and forward reference searches of included studies to identify additional references. The forward reference search was conducted until January 2021 using Web of Science to identify cited papers.

### 2.2. Study Selection Process

**Figure 1:**
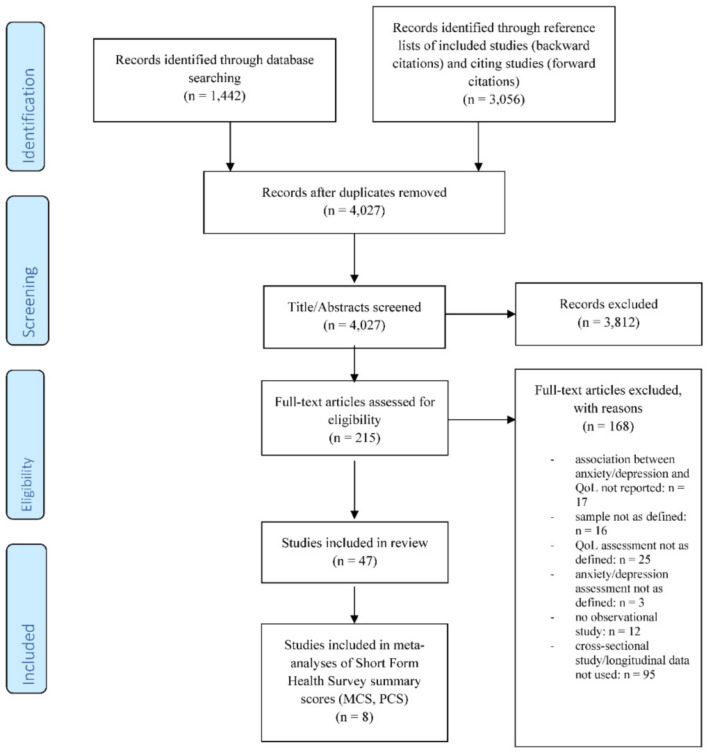
Study flow (PRISMA flow chart).

The study selection process is displayed in Figure 1. Most identified studies were screened in a two-step process (title/abstract; full-text screening) independently by two reviewers (J.K.H., E.Q.) against defined criteria (see Table 1). The last updated literature screening before submission was conducted by one reviewer (J.K.H.) and encompassed 9% of the studies included for title/abstract screening. Before the final criteria were applied, they were pretested and refined. Disagreements during the selection process were resolved through discussion or by the inclusion of a third party (A.H.) if a consensus could not be reached. 

### 2.3. Data Extraction and Synthesis

We extracted information regarding the study design, operationalization of the variables, sample characteristics, statistical methods and results regarding the research question of interest. If several analyses were presented for the same research question, we extracted the final covariate-adjusted model for narrative synthesis. Data were extracted by one reviewer (J.K.H.) and cross-checked by a second reviewer (E.Q.). If needed, extracted data were standardized (e.g., by calculating the weighted average means when combining groups) to present comparable information. If clarification was needed, the corresponding authors were contacted.

For the narrative synthesis, all studies were first grouped by research question, e.g., whether disorders or the degree of symptoms were analyzed, which comparison groups were used, which QoL domains were considered, and at which waves the variables of interest were considered in the analyses. Because research questions and analyses were heterogeneous, a concise narrative synthesis of the main results of all studies was not feasible. Therefore, we provide an overview of all identified studies in the tables and a detailed narrative synthesis of those studies, analyzing trajectories of disorders or changes in symptoms in association with changes in QoL over time.

Additionally, we examined whether data were appropriate for meta-analysis. The specific research questions, the operationalization of main variables and statistical methods were heterogeneous across studies and not all the statistical estimates needed could be obtained from covariate-adjusted analyses. Therefore, to enhance the comparability of the underlying data and the interpretation of the pooled estimates, we used descriptive information. Because most papers applied variations of the Short Form Health Survey and analyzed mental and physical component scores (MCS, PCS), we considered these studies as eligible for meta-analysis. The necessary information could be obtained for 8 publications. Random-effects meta-analysis was used for pooling. Heterogeneity was assessed by means of I^2^, with higher values representing a larger degree of heterogeneity in terms of variability in effect size estimates between studies [41]. Pooled estimates are reported as Hedge’s g standardized mean difference (SMD), representing the difference in mean outcomes between groups relative to outcome measure variability [42]. According to Cohen (as cited in [43]), SMDs can be grouped into small ≤0.20, medium = 0.50 and large effects ≥0.80. Stata 16 was used for meta-analyses.

### 2.4. Quality/Risk of Bias Assessment

Two reviewers (J.K.H., E.Q.) independently assessed the quality and risk of bias of the included studies using the Quality Assessment Tool for Observational Cohort and Cross-Sectional Studies, which was developed by the National Heart, Lung, and Blood Institute [44].

## 3. Results

### 3.1. Selection Process

The literature search yielded 4027 unique references. After title/abstract screening, 215 studies were included for full-text screening. Finally, 47 publications were included in the final synthesis. During full-text screening, most studies were excluded because they exclusively analyzed data on a cross-sectional level (56.5%). For further details, see the PRISMA flow chart (Figure 1).

### 3.2. Overview of Included Studies

Descriptive characteristics and quality/risk of bias assessment of the included studies are provided in Table S1 (Supplementary Material). In short, sample size ranged from 28 to 43,093. Most studies focused on adults; only four analyzed children/adolescents. Regarding the settings, 17 of the analyzed samples were exclusively recruited in a health care setting, 12 of the studies analyzed general population samples, 14 recruited in another or in several settings, and all studies on children/adolescents recruited in schools (*n* = 4). Twenty studies (42.6%) applied data from the same seven underlying datasets. Most studies reported on depression (*n* = 36), less reported on anxiety (*n* = 20) and some reported on the comorbidity between depression and anxiety (*n* = 7). To assess mental disorders, half (48.9%) used structured interviews. Regarding QoL, most studies applied variations of the Short Form Health Survey (SF, *n* = 27) or the WHOQOL (*n* = 12). A total of 38.3% of the studies were rated as “good”, 55.3% as “fair” and 6.4% as “poor” in the quality assessment.

### 3.3. Overview of Studies on the Association between Anxiety/Depression as Independent Variables and QoL Outcomes

Detailed results on all studies investigating the association between anxiety/depression as independent variables and QoL outcomes are reported in Table 2. As described in the methods section, the following paragraphs give an overview of those studies focusing on disorder trajectories/changes in symptoms over time and changes in QoL outcomes over time, because they allow for more differentiated interpretations.

**Depression as independent variable and QoL as outcome.** One study investigated QoL at several time points during the *entire course of an episode of MD*. 

Buist-Bouwman, Ormel, de Graaf and Vollebergh [46] analyzed an MD group from a general population setting (NEMESIS) with data on SF-36 domains in the onset, acute and recovery phase of the depressive episode. The onset of MD was associated with a significant drop in several QoL domains and recovery with a significant increase. Pre- and post-morbid QoL levels were not significantly different for most domains, and post-morbid QoL was even higher for the psychological role functioning and psychological health domains. In comparison to a group without MD, pre- and post-morbid QoL levels in the MD group were significantly lower, except for the psychological role functioning domain, where no significant differences were found. Additionally, it should be noted that 40% of the sample had lower post-morbid QoL compared to pre-morbid levels.

Two studies investigated changes in QoL for people experiencing an *onset of depression* relative to different comparison groups over two points in time.

One study investigated incident MD in a general population sample (NESARC; Rubio, Olfson, Perez-Fuentes, Garcia-Toro, Wang and Blanco [14]). Here, incident MD (compared to those without a history of MD as well as to a group without any mental disorder) was associated with a significant drop in QoL (SF-12 MCS). Additionally, analyzing two waves, Pyne, Patterson, Kaplan, Ho, Gillin, Golshan and Grant [67] compared the QoL (Quality of Well-Being scale) between MD patients and community controls. The patient group was further divided into those continuously not receiving an MD diagnosis, those who continuously received the diagnosis and those who only received the diagnosis at FU (onset). The authors found that changes in QoL did not differ between the groups. At both points in time, QoL scores differed significantly between the groups, except for the incident and the continuous depression group [67].

Six studies investigated different *courses of depression over time in people with depression at BL* with or without a healthy comparison group as reference.

Two primary care studies analyzed groups with clinical depression at BL with different FU depression statuses (remission, no remission). One study [51] analyzed changes in generic QoL measures (SF-12, WHOQOL-Bref) and the disease-specific Quality of Life in Depression Scale. In this study, remission was associated with an improvement in all QoL domains, whereas QoL did not change significantly over time for the non-remitted group. Another study [60] investigated SF-12 MCS and PCS scores and reported a significant increase in MCS over time in the remitting group. MCS scores in the continuously depressed group and PCS scores in both groups improved, albeit not significantly.

Another study [47] investigated whether chronic MD in a general population sample (NESARC) was associated with domain-specific reduced QoL (SF-12). They found that chronic MD was a significant risk factor for persistently reduced QoL in all domains and for the onset of reduced QoL at FU in all domains except for physical role.

Two population-based studies further differentiated between the depressive disorders. Analyzing MCS scores (NESARC), Rubio, Olfson, Villegas, Perez-Fuentes, Wang and Blanco [15] reported a significant increase in QoL for those who remitted from MD and from dysthymia relative to those who had a persistent disorder. Rhebergen, Beekman, de Graaf, Nolen, Spijker, Hoogendijk and Penninx [69] differentiated between people with MD, double depression or dysthymia at BL who remitted until FU relative to a group without a mental health diagnosis (NEMESIS). Physical health (SF-36) was lowest at BL for double depression, dysthymia and then the MD group. Over time, the MD and double depression groups improved significantly in their physical health, while the dysthymia group did not improve significantly. QoL was significantly lower relative to healthy comparisons for all depression groups at all waves. There were no significant differences regarding physical health trajectories over time among the depressive disorder groups.

Stegenga, Kamphuis, King, Nazareth and Geerlings [75] investigated more than two MD course groups over time (remitted, intermittent and chronic MD) in association with SF-12 MCS and PCS over time in a primary care-recruited sample with BL MD (Predict study). MCS increased over time in all groups, while changes in PCS were small. Compared to those who remitted, MCS at BL was significantly lower for the chronic course group. While the intermittent group also displayed a lower mean MCS at BL, the coefficient was not significant.

Three studies investigated *changes in depressive symptom levels* as the independent variable and changes in QoL as outcomes in adults.

One study found no significant association between an initial change in depressive symptoms and subsequent change in QoL (EQ-VAS) in older adults recruited in primary care [21]. The two other studies analyzed changes in depressive symptoms in samples with MD at BL [50,51]. Chung, Tso, Yeung and Li [50] found that changes in depressive symptom levels was associated with changes in several QoL domains (SF-36: general health, vitality, social functioning, mental health and MCS). Diehr, Derleth, McKenna, Martin, Bushnell, Simon and Patrick [51] investigated whether quartiles of change in depressive symptoms were associated with changes in QoL (SF-12, QLDS and WHOQOL-Bref). Those without any change in depressive symptoms generally showed no change in QoL. For all QoL domains and scores except for SF-12 PCS, improvement in depressive symptoms over time was associated with a significant increase in QoL, while a reduction in depressive symptoms was associated with a significant reduction in QoL. Those who had the largest reduction in depressive symptoms also had the largest improvement in QoL measures.

**Anxiety as an independent variable and QoL as an outcome.** Two publications used a general population sample (NESARC) to investigate *incident anxiety disorders* [14] and the *remission of anxiety disorders* [15] in association with SF-12 MCS. Both studies separated generalized anxiety disorder (GAD), social anxiety disorder (SAD), panic disorder (PD) and social phobia (SP). All incident disorders were associated with a significant reduction in QoL compared to people without a history of the specific disorders. When the analysis was restricted to incident cases without comorbidities, QoL levels were not significantly different compared to people without a history of any psychiatric disorder [14]. Those who remitted from SAD showed a significant increase in QoL compared to persistent cases. While QoL improved for all remitting anxiety disorders, change scores for PD and SP were not significant [15].

Another study investigated *different courses* (intermittent, chronic or remitting) of obsessive compulsive disorder (OCD) and course in QoL (EQ-5D) as well as a comparison group from the general population [68]. They found that the OCD groups mostly reported a lower QoL compared to the general population. Moreover, the course groups differed regarding their QoL over time, with remitters reporting small to moderate improvements compared to the chronic group.

One study investigated changes in *anxiety symptoms* in association with changes in all SF-36 domains and both summary scores over time in a sample with MD at BL [50]. Changes in anxiety symptoms were significantly associated with changes in bodily pain, general health and the mental health domain.

### 3.4. Overview of Studies on the Association between QoL as Independent Variable and Anxiety/Depression as Outcomes

Additionally, we identified publications operationalizing QoL as the independent variable and anxiety/depression as outcomes with details on all studies reported in Table 3. Only one study reported on change in QoL over time and change/trajectories in mental health outcomes over time. This study operationalized change in QoL as a predictor of future change in depressive symptoms over time and reported that an initial improvement in EQ-VAS was associated with a future reduction in depressive symptoms in older adults [21].

### 3.5. Meta-Analyses on Anxiety, Depression and SF Summary Scores 

In total, eight studies on adults were included in a supplementary meta-analyses of several research questions on SF PCS and MCS in association with anxiety and depressive disorders. Forest plots for the analyses are provided in the supplementary materials (Figures S1–S10).

**Differences in SF summary scores at FU among adults with and without depressive disorders at BL.** Based on a pooling of four studies [45,49,52,54], those with depression at BL showed lower MCS scores at FU compared to a group without depression at BL with a large effect size (SMD = −0.96, 95% CI: −1.04 to −0.88, *p* < 0.001, I^2^ = 0.0%). PCS scores at FU were lower for the depression group compared to the non-depression group with a medium effect size (SMD = −0.68, 95% CI: −1.06 to −0.30, *p* < 0.001, I^2^ = 94.6%). Excluding the study rated “poor” in the quality/risk of bias assessment from the pooling did not substantially affect the results (MCS: SMD = −0.96, 95% CI: −1.03 to −0.88, *p* < 0.001, I^2^ = 0.01%; PCS: SMD = −0.63, 95% CI: −1.08 to −0.19, *p* < 0.01, I^2^ = 96.8%).

**BL differences in SF summary scores among adults with MD at BL with and without remitting courses over time.** Based on a pooling of two studies [19,84] of samples with MD at BL, those with persistent MD at FU had significantly lower MCS at BL (SMD = −0.25, 95% CI: −0.41 to −0.10, *p* = 0.001, I^2^ = 74.95) and PCS scores at BL (SMD = −0.24, 95% CI: −0.39 to −0.09, *p* = 0.002, I^2^ = 73.14) compared to those who achieved remission until FU. Effect sizes were small for both summary scores.

**FU differences in SF summary scores among adults with depressive and anxiety disorders at BL with and without remitting courses**. Based on the pooling of two studies [71,81] of samples with MD and/or dysthymia, the group where the disorder had persisted/a co-morbid condition was present/had a suicide attempt until FU had significantly lower MCS scores at FU compared to the group where the disorder had remitted without treatment until FU, with a medium effect size for depressive disorders (SMD = −0.59, 95% CI: −0.75 to −0.42, *p* < 0.001, I^2^ = 37.72) and a small effect size for anxiety disorders (SMD = −0.44, 95% CI: −0.58 to −0.30, *p* < 0.001, I^2^ = 58.87). The SMD for PCS scores at FU was negligible in terms of effect size for both disorder groups (depressive disorders: SMD = 0.02, 95% CI: −0.24 to 0.27, *p* = 0.90, I^2^ = 73.65; anxiety disorders: SMD = −0.09, 95% CI: −0.17 to −0.01, *p* = 0.03, I^2^ = 0.01).

## 4. Discussion

### 4.1. Main Results

This review adds to the present literature by providing an overview of longitudinal observational studies investigating the association between depression, anxiety and QoL in samples without other specific illnesses or specific treatments. Additional meta-analyses investigated group differences according to SF MCS and PCS.

While a concise synthesis of all the identified studies is challenging due to heterogeneity, the following picture emerges from studies investigating change–change associations: before the onset of disorders, QoL is already lower in disorder groups in comparison to healthy comparisons. The onset of the disorders further reduces the QoL. Remission is associated with an increase in QoL, mostly to pre-morbid levels. Additionally, some studies show that remission patterns are relevant for QoL outcomes as well. Moreover, a bi-directional effect was reported, whereby QoL is also predictive of mental health outcomes.

Evidence for a bi-directional association as well as studies showing lower QoL across the entire course of the disorders (before onset, during disorder, after disorder) relative to a healthy comparison group seem to suggest that impairments in QoL may result from a certain pre-disorder vulnerability in these groups. Longitudinal studies using general population data have investigated different hypotheses on (QoL) impairments after remission of anxiety disorders and MD [87,88]. One hypothesis suggests that impairments after the illness episode reflect a pre-disorder vulnerability (vulnerability or trait hypothesis), while the another states that impairments develop during the mental health episode and remain as a residual after recovery (scar hypothesis). Generally, both studies favored the vulnerability hypothesis [87,88]. For subgroups with recurrent anxiety disorders, scarring effects were also found for mental functioning [88]. Yet, it has to be noted that it was not the aim of our review to gather evidence for these hypotheses using QoL as an indicator, which represents an opportunity for future research.

To be able to investigate possible domain-specific differences across studies, we aimed to conduct a meta-analysis on all studies on the same research question which reported on QoL subdomains (e.g., using WHOQOL and SF). However, as described in the Methods section above, only eight studies reported comparable information on different research questions and could be included in meta-analyses. Due to the limited number of studies included in each meta-analysis, the focus on SF MCS and PCS scores, and most studies reporting on depression, the results of the meta-analyses should be viewed with caution. Keeping this in mind, our results indicate that both mental and physical QoL are significantly impacted by anxiety and depressive disorders and that the course of the disorder is also relevant for QoL outcomes. Not surprisingly, effect sizes for MCS were larger compared to PCS for most research questions. A pooling of two studies on different courses of anxiety and depressive disorders found that effect sizes for MCS at FU were of moderate size for depressive (SMD = −0.59) and of small size for anxiety disorders (SMD = −0.44), while SMDs for PCS at FU were negligible in size.

Overall, effect sizes from meta-analyses ranged from negligible to large, and heterogeneity varied considerably (I^2^ between 0% and 95%). Because of the small number of studies, possible influential study-level factors (e.g., setting, operationalization of the variables, length of FU) could not be investigated in further detail by means of a meta-regression, which remains a question for future research.

### 4.2. Implications for Future Research

Based on the results described and study heterogeneity discussed above, we provide recommendations for future research.

First recommendation: future research should differentiate between individual disorders and focus on anxiety disorders. The majority of the studies investigated depressive disorders or symptoms. On the level of individual disorders, most focused on MD, while two studies additionally reported on dysthymia [15,69]. One of these investigated double depression [69]. On the level of anxiety disorders, three publications differentiated between individual anxiety disorders within the same study [14,15,63]. While it was not possible to conduct a meta-analysis comparing different anxiety disorders in our case, individual studies suggest possible disorder-specific differences when analyzing changes in QoL over time: Rubio, Olfson, Villegas, Perez-Fuentes, Wang and Blanco [15] suggest that QoL significantly improved for those remitting from GAD and SAD (compared to non-remission). QoL improved for PD and SP as well, but differences in change scores were smaller and did not reach statistical significance. The incidences of all of these disorders were associated with a significant drop in QoL [14]. In summary, future longitudinal studies should focus on anxiety disorders and generally differentiate between individual disorders to investigate possible disorder-specific differences.

Second recommendation: future research should consider trajectories of disorders/change in symptoms and changes in QoL over time. We would have liked to include a meta-analysis of disorder trajectories and change scores in QoL over time. Because of the small, diverse number of studies on this association in general and the number of assumptions that would have had to have been made for a meta-analysis, we refrained from pooling effects for this research question. In total, 17 studies investigated changes in independent variables associated with changes in outcomes. This approach has several advantages. On the one hand, different disorder or symptom trajectories can be identified. Several studies reported that QoL outcomes differ according to disorder course and the degree of change in symptoms. The focus on the change in characteristics over time in future research could additionally reduce the problem of unobserved time-constant heterogeneity in observational studies when appropriate methods are applied [26].

Third recommendation: future research should investigate individual QoL domains. Several systematic reviews on cross-sectional studies found that effect sizes differed by QoL domains [32,89]. For example, Olatunji, Cisler and Tolin [89] reported that health and social functioning were most impaired for anxiety disorders (compared to non-clinical controls). Comparing individuals with diabetes and depressive symptoms to those with diabetes only, Schram, Baan and Pouwer [32] reported that while SF pain scores were mild to moderately impaired, role and social functioning displayed moderate to severe impairments in those with comorbid depressive symptoms. The other scores were moderately impaired. As described above in detail, a meta-analysis using all subdomains was not feasible in this review. Further research differentiating between QoL domains would thus allow future meta-analyses to investigate whether the observed domain-specific differences reported in previous reviews of cross-sectional data can be observed in longitudinal studies as well.

Fourth recommendation: future research should consider bi-directional effects. While investigating QoL as the outcome measure and anxiety/depression as independent variables seems relatively straightforward, ten studies investigated QoL as the independent variable and anxiety/depression as outcomes. In light of possible bi-directional effects and pre-existing vulnerability suggested by individual studies, future research considering QoL as an independent variable could inform a deeper understanding of this complex association. 

### 4.3. Strengths and Limitations

A strength of this work is the transparent methodological process: the review was prospectively registered with PROSPERO and a study protocol was published [34]. Two reviewers were included in screening, data extraction and quality assessment processes. There were no limitations regarding the time or location of the publications. Moreover, all versions of the ICD/DSM and validated questionnaires were considered eligible to identify anxiety or depression. Another strength is the thorough literature search that enabled us to identify all relevant studies. Additionally, we did not limit the age range and were therefore able to shed light on studies investigating children/adolescents. Moreover, some studies could be pooled using random-effects meta-analyses, which allows for stronger conclusions regarding effect sizes compared to individual studies. Besides the content analysis, this review emphasizes difficulties in meta-analysis from observational, longitudinal studies. We hope that our work can facilitate discussion on this topic.

The study has some limitations. We did not limit our search to specific research questions, which led to the inclusion of heterogeneous studies. Heterogeneity particularly stemmed from the operationalization of the variables of interest. Due to this, a concise narrative synthesis of all results was not feasible. The positive aspect of this broad focus is that it allowed us to provide an overview of studies and research questions analyzed and to formulate more nuanced recommendations for future research. We have to acknowledge that there is an abundance of QoL assessments used in medicine and health sciences [37]. The list applied in this work was derived with respect to previous relevant reviews on QoL research. It was not designed to be fully comprehensive or exhaustive. Rather, it provided us with a working definition for this review and helped to enhance the transparency of our selection processes. Additionally, because we included validated QoL measures frequently used in research, we assume that exclusion would particularly have been the case for novel or study-specific measures. Finally, the focus on peer-reviewed literature means that studies in other languages and gray literature were not considered. Nonetheless, this focus on literature published in peer-reviewed journals should ensure a certain scientific quality.

## 5. Conclusions and Relevance for Clinical Practice

Overall, the results indicate that QoL is lower before the onset of anxiety and depressive disorders, further reduces upon onset of the disorders and generally improves with remission to pre-morbid levels. Moreover, disorder course (e.g., remitted, intermittent, chronic) seems to play an important role; however, only a few studies analyzed this. Changes in anxiety and depressive symptoms were also associated with changes in QoL over time. Meta-analyses found that effect sizes were larger for MCS relative to PCS, highlighting the relevance of differentiation between QoL domains. While our review identified some gaps in the current literature and made recommendations for future research, the following should be noted for clinical practice. On the one hand, an improvement in mental health is associated with better QoL, which emphasizes the relevance of support during the disorders. This is also shown by meta-analyses, which show that cognitive behavioral therapy additionally improves QoL [90,91]. Moreover, the results indicate reduced QoL even before disorder onset, highlighting the relevance of early preventive measures in vulnerable groups. In line with this, studies on school-based prevention programs show a significant reduction in anxiety and depressive symptoms [92,93], and psychosocial prevention programs may additionally improve QoL [94]. 

During the COVID-19 pandemic, it is of high relevance to tackle the arising challenges associated with this pandemic. For example, it is important to face the high prevalence rates of both depression and anxiety with appropriate measures.

## Figures and Tables

**Table 1:** Study selection criteria.

**Inclusion criteria**	Studies were eligible for inclusion if they: (i) Were observational studies analyzing the longitudinal association between anxiety or depression (disorders as well as symptom severity) and QoL, (ii) Analyzed samples without a specific disease or disorder other than anxiety and depression, (iii) Applied appropriate, validated measures for the main variables (e.g., for anxiety/depression: psychiatric diagnosis according to criteria of the International Classification of Diseases (ICD), the Diagnostic and Statistical Manual of Mental Disorders (DSM), or using a valid self-report screening tool), and (iv) Were published in English or German in a peer-reviewed journal.
**Exclusion criteria**	Studies were excluded if they: (i) Analyzed samples where participants were suffering or recovering from conditions other than anxiety/depression, (ii) Analyzed samples receiving or recovering from a specific intervention or treatment, (iii) Had no observational study design, (iv) Used a measure for the main variables other than those defined, or (v) Had publication characteristics that were different than those defined (e.g., were published in a language other than German or English, as well as not published in a scientific, peer-reviewed journal).
**Refinements after pre-tresting and reason for refinement**	After pre-testing, the following refinements were made to the screening criteria (ii) and (iii): (ii) Regarding the samples of interest, we decided to exclude studies analyzing dyads such as caregivers or partners to ill family members, due to possible spillover-effects on the individual’s QoL, which has been demonstrated in previous studies [35,36]. Additionally, samples consisting exclusively of people with anxiety or depressive disorders may receive some unspecific type of care for their mental health problems. We eliminated studies evaluating the effects of treatments using pre–post-treatment comparisons. Only studies where some naturalistic treatment that is usual for mental health problems that began prior to study baseline (BL) were included. Studies indicating that treatment was initiated at or after study BL (e.g., before or at admission to a psychiatric clinic) were excluded. (iii) Lastly, we specified the QoL assessments. In health and medicine research, numerous QoL instruments are used [37]. Guided by previous literature reviews [38,39,40]), we compiled a list of ten validated QoL assessments that have been used in children, adolescents or adults from the general population and/or samples with mental health problems, and that are frequently used in QoL research. Versions of the following instruments were included: Short Form Health Survey (e.g., SF-36, SF-12), EuroQol (e.g., EQ-5D, EQ-5D-Y), WHOQOL (e.g., WHOQOL-100, WHOQOL-BREF), Quality of Well-Being Scale, Quality of Life Scale, Pediatric Quality of Life Inventory, KIDSCREEN, KINDL, Quality of Life in Depression Scale, and the Quality of Life Enjoyment and Satisfaction Questionnaire.

Abbreviations: QoL = quality of life; ICD = International Classification of Diseases; DSM = Diagnostic and Statistical Manual of Mental Disorders; BL = study baseline; KIDSCREEN = Health Related Quality of Life Questionnaire for Children and Young People and their Parents; KINDL = German generic quality of life instrument for children

**Table 2:** Studies on depression/anxiety as independent variables and QoL outcomes.

First Author (Year)	Disorder or Symptoms Analyzed; QoL Domains Analyzed	Research Question Regarding QoL	Methods	Results
Årdal (2013) [45]	Controls and patients in the acute phase of recurrent MD and FU (DSM-IV, HDRS); SF-36 (physical functioning, role physical, vitality, bodily pain, mental health, role emotional, social functioning, general health, as well as summary scores PCS, MCS and total score)	(a) Whether QoL scores differ between MD patients and healthy comparisons across domains over time. (b) Whether QoL in patients with recurrent MDD differed between acute phase and recovery.	(a) ANOVA (b) Paired-sample *t*-tests	(a) There was a significant interaction effect between time, QoL domain and group, indicating that QoL scores differed between MD patients and controls over time. Compared to the healthy control group, the MDD group had reduced QoL in all domains at BL and reduced QoL in several domains at FU (significant for general health, social, emotional role, mental health, PCS, MCS and total score). (b) In the MD group, QoL scores significantly improved during recovery from recurrent MDD in most domains (significant for physical functioning, physical role, vitality, social functioning, role emotional, mental health, PCS, MCS and total score).
Buist-Bouwman (2004) [46]	Onset, acute phase and subsequent remission from MDE (CIDI); comorbid anxiety disorder (CIDI); SF-36 (physical functioning, physical role, vitality, pain, psychological health, psychological role, social functioning and general health)	(a) Whether incident MDE and recovery from MDE are associated with changes in QoL and whether pre- and post-morbid QoL scores in the MD group differ from the comparison group without MD. (b) In the subgroup with worse QoL after MDE: whether the severity of depression and number of depressive episodes were associated with worse QoL. (c) Whether comorbid anxiety during MDE is associated with reduced QoL (i.e., lower QoL after MDE compared to before MDE).	(a)–(c) Multivariate logistic regression	(a) Incident MDE was associated with a drop in QoL (significant for vitality, psychological, psychological role and social functioning). Subsequent recovery from MDE was associated with an improvement in QoL (significant for physical role, vitality, psychological health, psychological role, social functioning and general health). Comparing pre- and post-morbid levels, QoL did not differ or was higher after MDE in some domains (significantly higher for psychological health and psychological role). Moreover, before MD onset, QoL was significantly lower compared to healthy controls in all domains. After remission from MDE, QoL scores in nearly all domains (not significant for psychological role) were significantly lower compared to healthy controls. (b) About 40% of the MDE group had worse QoL after recovery from MDE compared to pre-morbid levels. The severity of depression was associated with worse QoL only for the psychological health domain, but no other domains. The number of depressive episodes was not significantly associated with worsening QoL in any domain. (c) In the MDE cohort, comorbid anxiety was associated with a significant reduction in QoL (significant for physical role and psychological health).
Cabello (2014) [47]	Chronic MD (AUDADIS interview; summary score of the number of symptoms to identify severity); SF-12, “disability” (i.e., domain-specific reduced QoL, defined as score ≤ 25th percentile in the subscale; physical functioning, physical role, bodily pain, general health, vitality, social functioning, emotional role and mental health)	(a) Whether chronic MD is associated with the incidence/persistence of “disability” (i.e., reduced QoL) in a general population sample. (b) Whether the severity of depressive symptoms is associated with the incidence/persistence of “disability” (i.e., reduced QoL) in the MD subgroup of the sample.	Both (a) and (b) Generalized Estimating Equations and logistic regressions	(a) In the general population, chronic MD was a significant risk factor for the persistence of disability (i.e., reduced QoL) in all domains and of the incidence of disability (i.e., reduced QoL) in all domains except for the physical role. (b) In the chronic MD subgroup, the severity of depressive symptoms was associated with the persistence of disability (i.e., reduced QoL) (significant for general health, social functioning, emotional role and mental health) and not significantly associated with the incidence of reduced QoL in any domain.
Cerne (2013) [48]	Number of depressive episodes over time according to CIDI; number of episodes of panic and other anxiety syndromes over time (PHQ); SF-12 (PCS, MCS)	Whether the pooled number of (a) depressive episodes over time, (b) panic and anxiety episodes over time are are associated with the pooled QoL over time.	(a) and (b) Multivariate linear regression	(a) A higher number of depressive episodes over time was associated with lower pooled PCS and MCS. (b) a higher number of pooled panic episodes over time was associated with a lower mean MCS but not PCS. A higher number of pooled other anxiety syndrome episodes over time was not associated with the mean MCS or PCS.
Chin (2015) [49]	Depression according to PHQ-9 (>9), clinician’s diagnosis; SF-12v2 (PCS, MCS)	(a) Whether depressive symptoms and a clinician’s detection of depression at BL are associated with QoL at FU. (b) Whether a clinician’s detection of depression at BL is associated with a change in QoL.	(a) Multivariable non-linear mixed-effects regression (b) Independent *t*-tests	(a) Depressive symptoms and a clinician’s detection of depression at BL were not predictive of QoL at FU. (b) A clinician’s detection of depression at BL was related to change (improvement) in MCS, but not PCS over time in a primary care sample screened as positive for depression.
Chung (2012) [50]	Depression diagnosis and symptoms (DSM-IV, HRSD_17_ depression scale, HADS depression scale); anxiety symptoms (HRSD_17_ anxiety scale, HADS anxiety scale; SF-36 (physical functioning, role physical, bodily pain, general health, vitality, social functioning, role emotional, mental health, PCS and MCS)	(a) Whether BL depressive symptoms are associated with QoL at FU. (b) Whether BL depressive symptoms or changes in depressive symptoms are associated with changes in QoL over time. (c) Whether BL anxiety symptoms are associated with QoL at FU. (d) Whether BL anxiety symptoms or changes in anxiety symptoms are associated with changes in QoL over time.	(a)–(d) Hierarchical regression	(a) BL depressive symptoms were not associated with any QoL domain at FU. (b) BL depressive symptoms were not associated with changes in any QoL domain over time. Changes in depressive symptoms were significantly associated with changes in some QoL domains over time (significant for: general health, vitality, mental health and MCS). (c) BL anxiety symptoms were not associated with any QoL domain at FU. (d) BL anxiety symptoms were not associated with changes in any QoL domain over time. Changes in anxiety symptoms were significantly associated with changes in some QoL domains over time (significant for: bodily pain, general health and mental health).
Diehr (2006) [51]	Depression according to CIDI, CES-D (>16); QLDS, WHOQOL-Bref (environmental, physical, psychological and social), SF-12 (PCS, MCS)	(a) Whether the quartile of change in depressive symptoms is associated with changes in QoL. (b) Whether the remission of depression at FU is associated with changes in QoL.	Regression	(a) No/little change in CES-D associated with changes in QoL over time (significant for SF-12 MCS). Every other quartile of change in depressive symptoms was significantly associated with changes in QoL in most scales/domains (significant for: QLDS, all domains of WHOQOL-Bref and SF-12 MCS), meaning a higher reduction in depressive symptoms was associated with a higher increase in QoL, and more severe depressive symptoms were associated with a reduction in QoL. (b) Remission of depression at FU was associated with improvement in all QoL measures and domains (SF-12, QLDS and WHOQOL-Bref). There was no significant change in QoL in those with persistent clinical depression at FU.
Hajek (2015) [21]	Depressive symptoms (GDS); EQ-VAS	Whether an initial change in depressive symptoms is associated with a subsequent change in QoL in the whole sample and by sex.	Vector autoregressive models	No significant association between an initial change in depression score and a subsequent change in QoL was found for the whole sample or stratified by sex.
Hasche (2010) [52]	Depression status at BL (according to DIS diagnosis and CES-D ≥ 9); SF-8 (PCS, MCS)	(a) Whether depression status groups at BL differed according to QoL at FU. (b) Whether depression status groups at BL differed according to QoL changes in score over time.	(a) *t*-tests (b) Linear mixed effects regression models	(a) At 6- and 12-month FU, those with and without depression at BL differed significantly in QoL scores, with the depression group reporting lower QoL at FUs (significant for MCS and PCS). (b) While depression at BL was significantly related to improvements in MCS (but not PCS) scores over time, those with depression still reported lower QoL compared to those without.
Heo (2008) [53]	Depression (BDI ≥ 10); SF-36 (decrease in total score over time)	Whether FU depression is associated with a reduction in QoL over time.	Binary logistic regression	Depression at FU was associated with a significant reduction in QoL total score over time.
Ho (2014) [54]	Depression (according to GDS ≥ 5); SF-12 (PCS, MCS)	Whether depression at BL is associated with QoL at FU.	Linear regression	BL depression was associated with lower QoL at FU (significant for MCS and PCS).
Hussain (2016) [55]	Depressive disorders (SCID, MINI); current PTSD, specific phobias, other anxiety disorders (SCID, MINI); WHOQOL-Bref (general QoL and hrqol)	(a) Whether current depressive disorders at BL predict QoL at FU. (b) Whether current PTSD, specific phobias and other anxiety disorders at BL predict QoL at FU.	(a) and (b) Multiple linear regression	(a) Depressive disorders at BL predicted reduced QoL at FU (significant for general QoL and hrqol). (b) PTSD, but not specific phobias or other anxiety disorders, predicted reduced general QoL at FU. None of the anxiety disorders predicted hrqol at FU.
Joffe (2012) [56]	Lifetime history of depression (according to SCID); anxiety disorder (according to SCID); SF-36 (impaired QoL according to 25th percentile of SF-36; social functioning, role emotional, role physical, pain and vitality)	(a) Whether a lifetime history of depression is associated with impaired QoL during FU. (b) Whether a prior lifetime history of anxiety disorder (compared to no depression or anxiety) is associated with reduced QoL during FU. (c) Whether a lifetime history of comorbid depression and anxiety is associated with impaired QoL during FU.	(a)–(c) Repeated measure multilevel regression	(a) A history of depression only was associated with reduced QoL during FU (significant for social functioning and pain). (b) Prior lifetime history of anxiety disorder was associated with reduced QoL (significant for physical role). (c) A history of comorbid anxiety and depression was associated with reduced QoL during FU (significant for social functioning, emotional role, physical role and pain).
Johansen (2007) [57]	Level of PTSD symptoms according to IES-15; WHOQOL-Bref (physical health, psychological health, social relationships and environment)	Whether PTSD symptoms predict QoL at FU.	Structural equation model	More severe PTSD symptoms predicted QoL at FU (significant positive association between FU1 and FU2).
Kramer (2003) [58]	Current or lifetime depression/PTSD (according to Q-DIS); SF-36 (energy/fatigue, emotional role, general health, mental health, pain, physical functioning, physical role and social)	Whether QoL outcomes over time differed among the disorder groups.	Random/fixed effects model	There was no significant interaction between time and diagnostic group (no depression/PTSD, PTSD, depression and comorbid depression/PTSD) on QoL. Comparing the adjusted means for all three times among the disorder groups showed significant differences between the groups in most domains. In comparison, those with depression at BL reported reduced QoL over time in several domains compared to the PTSD group and the group without PTSD/depression. In comparison, those with PTSD only showed higher QoL compared to those with depression or comorbid depression/PTSD in several domains.
Kuehner (2009) [20]	Depressive symptoms (MADRS); WHOQOL (overall, physical, psychological, social and environmental)	Whether the lag in levels of depressive symptoms predicts future levels of QoL and whether the association differs by group (formerly depressed inpatients vs. community controls).	Time-lagged linear models	Higher depressive symptoms predict future lower QoL (significant for social). The association was not moderated by group status.
Kuehner (2012) [59]	Depression score (according to MADRS, FDD-DSM-IV); WHOQOL-Bref (physical, psychological, social and environment)	Whether the lag in depressive symptoms predicted QoL at FU.	Hierarchical, time-lagged linear models	Higher depressive symptoms significantly predicted lower QoL at FU (significant for physical and psychological).
Lenert (2000) [60]	Remission or persistent depression (according to DSM-III criteria, DIS); SF-12 (PCS, MCS)	Whether the remission of depression (compared to no remission) is associated with changes in QoL over time.	OLS regression	Remission of depression was associated with improved QoL (significant for MCS) at FU1 and FU2.
Mars (2015) [61]	Asymptomatic, mild and high symptoms of depression (according to SCAN); EQ-5D (without anxiety/depression item)	Whether depression symptom trajectories over time (asymptomatic, mild symptoms and chronic–high symptoms) are associated with QoL at FU.	Latent class growth analysis with distal outcome models	QoL at FU differed significantly among different depression symptom trajectories, with persons from the the chronic–high depressive symptom class showing lower QoL scores relative to the asymptomatic class.
Moutinho (2019) [62]	Depression at BL (according to DASS cut-off: 9); anxiety at BL (according to DASS anxiety scale cutoff: 7); WHOQOL-Bref at FU (physical, psychological, social and environment)	(a) Whether BL depression predicted QoL at FU. (b) Whether BL anxiety predicted QoL at FU.	(a) and (b) Stepwise linear regression	(a) Depression at BL was significantly associated with reduced QoL at FU (significant for psychological functioning, social functioning and environmental). (b) Anxiety at BL was associated with reduced QoL at FU (significant for physical).
Ormel (1999) [63]	Depression at BL (according to CIDI); “disability” (i.e., reduced QoL according MOS SF 6-item physical functioning scale ≥ 2)	Whether depression at BL is associated with the onset of disability (i.e., reduced QoL) during FU.	Logistic regression models	Compared to the non-depressed group, people with depression at BL showed higher odds for the onset of disability (i.e., reduced QoL) during FU (significant for 12-month FU, but not 3-month FU).
Pan (2012) [64]	Depressive symptoms (CES-D); WHOQOL-Bref-TW (overall score, physical, psychological, social and environmental)	Whether depressive symptoms were associated with QoL over time.	Linear mixed-effects models	Higher depressive symptoms were associated with lower QoL in MDD patients (significant for overall score, physical, psychological, social and environmental).
Panagioti (2018) [65]	Depressive symptoms (MHI-5); WHOQOL-Bref (physical, psychological, environmental and social)	Whether depressive symptoms at BL are associated with changes in QoL over time.	Multivariate regression models	Higher depressive symptoms at BL were associated with a decline in QoL over time (significant for physical and psychological).
Pakpour (2018) [66]	Dental anxiety at BL (MDAS); PedsQL 4.0 general hrqol and oral hrqol scale at FU	Whether dental anxiety at BL predicted oral- and general-health-related QoL at FU.	Structural equation modeling	Dental anxiety at BL was no significant direct predictor of generic QoL at FU and was significantly associated with worse oral-health-related QoL at FU.
Pyne (1997) [67]	MD-diagnosis (SCID/SADS) and depressive symptoms (HAM-D); QWB	Whether group status over time (community controls, continuously non-depressed patients, incident depression patients and continuously depressed patients) is associated with changes in QoL.	Repeated measure analysis (ANOVA)	There was no significant interaction term between group status and time, indicating that changes in QoL did not differ between the groups. At both points in time, QoL differed significantly among all groups, except between the incident depression and continuous depression group.
Remmerswaal (2020) [68]	OCD course (SCID), Y-BOCS, BDI, BAI over time; EQ-5D over time	(a) Whether OCD symptom severity and QoL over time were associated. (b) Whether QoL over time differs between OCD course groups (chronic, intermittent and remitting) and general population norms. (c) Whether OCD symptom severity, anxiety and depressive symptoms over time are associated with changes in QoL over time in patients with OCD.	(a) Pearson’s correlation (b)–(c) Linear mixed models	(a) QoL over time and OCD symptom severity were significantly correlated. (b) The QoL of OCD patients was significantly lower compared to general population norms, except the QoL of the intermittent OCD group at FU1, where there was no significant difference compared to the general population. When comparing the OCD course groups, the chronic OCD group had a significantly lower QoL over time compared to the other groups. The remitting group had moderately improved until FU1 and a small QoL improvement between FU1 and FU2 relative to the chronic group. (c) In those with a remitting OCD, only more severe symptoms of comorbid anxiety and depressive symptoms, but not OCD symptom severity over time, were significantly associated with a lower QoL over time.
Rhebergen (2010) [69]	MD-/dysthymia-/DD diagnosis at BL and subsequent recovery at FU (according to CIDI); comorbid anxiety at BL (CIDI); SF-36 (physical health summary score)	Whether QoL trajectories over time differ between: (a) different depression status groups who achieved remission (MDD, dysthymia and double depression) and a comparison group without mental health disorders. (b) The different depression status groups. (c) Whether comorbid anxiety at BL in a sample recovering from depression is associated with changes in QoL.	(a)–(c) Linear mixed models	(a) There was a significant interaction between group status and time. More specifically, compared to changes in QoL over time in people without a mental health diagnosis, QoL improved over time in those with MDD and DD, but not dysthymia. All depression diagnosis groups showed a significantly lower QoL compared to the no diagnosis group at all waves. (b) Considering the depression groups, only the interaction term between dysthymia and time until FU1 was significant. Those with dysthymia had a significantly lower QoL compared to those with MDD at FU1. This difference was not significant at FU2. (c) Comorbid anxiety disorder at BL in people who recovered from depression over time was not associated with a significant change in QoL over time.
Rubio (2014) [14]	First episode of incident MDD (AUDADIS-IV) at FU; incident GAD, social anxiety disorder, PD, specific phobia (AUDADIS-IV); SF-12 (MCS)	Whether incident MDD is associated with changes in QoL over time compared to: (a) people without history of MDD, (b) without history of any mental health disorder, (c) and whether the association differed by gender. Whether incident anxiety disorders are associated with changes in QoL over time: (d) compared to no history of the specific anxiety disorder, (e) compared to no history of any psychiatric disorder, (f) and whether the association differed by gender.	Linear regression model	(a) Incidence of MDD (compared to no MDD) was associated with a significant decrease in QoL until FU. (b) Incidence of MDD (compared to no mental health disorder) was associated with a significant decrease in QoL until FU. (c) The association did not vary by gender. (d) Incidence of all anxiety disorders (with comorbid disorders; ref: no history of anxiety disorder) was associated with a significant decrease in QoL over time. (e) Incident anxiety disorders were not significantly associated with QoL when only considering “pure” anxiety without any comorbidities (ref: no history of any psychiatric disorder). (f) The association did not vary by gender.
Rubio (2013) [15]	Remission from MDD, dysthymia (AUDADIS-IV); Remission from GAD, PD, SAD, specific phobia (AUDADIS-IV); SF-12 (MCS)	Whether remission from depression (MDD, dysthymia) is associated with: (a) changes in QoL over time (compared to non-remitted cases), (b) QoL at FU (compared to people with no history of a specific depressive disorder), (c) QoL at FU, when only considering depressive disorders without any psychiatric comorbidity (compared to people without any lifetime psychiatric diagnosis). Whether remission from anxiety disorders are associated with: (d) changes in QoL over time (compared to non-remitted cases), (e) QoL at FU (compared to people with no history of a specific anxiety disorder), (f) QoL at FU, when only considering anxiety disorders without any psychiatric comorbidity (compared to people without any lifetime psychiatric diagnosis).	(a)–(f) Linear regression models	(a) Remission from MD and dysthymia was associated with a significant positive change in QoL compared to non-remitted cases. (b) Remission of MD and dysthymia was associated with significantly lower QoL at FU compared to people without history of a specific diagnosis. (c) Remission of MD and dysthymia was associated with significantly lower QoL at FU compared to people without any lifetime psychiatric diagnosis. (d) Remission from SAD and GAD was associated with significant positive changes in QoL compared to non-remitted cases. (e) Remission of PD, SAD, specific phobia and GAD was associated with significantly lower QoL at FU compared to people without history of a specific diagnosis. (f) Remission of “pure” PD, SAD, specific phobias and GAD was associated with significantly lower QoL at FU compared to people without any lifetime psychiatric diagnosis.
Rozario (2006) [70]	Depressive symptoms (GDS); SF-12 (MCS and PCS)	Whether depressive symptom severity was associated with QoL change profiles over time (no change, declined and improved groups).	Multinomial logistic regression	There was no significant association between depressive symptom severity and QoL change score profiles at FU.
Sareen (2013) [71]	Depression trajectory groups over time (according to AUDADIS-IV); anxiety disorder trajectory groups over time (according to AUDADIS-IV); SF-12 (MCS and PCS)	(a) Whether depression trajectory groups (no past year disorder/no suicide attempt at FU, remission without treatment, persistent disorder/comorbidity/suicide attempt/treatment) differed according to QoL at FU. (b) Whether anxiety disorder trajectory groups (no past year disorder/no suicide attempt at FU, remission without treatment, persistent disorder/comorbidity/suicide attempt/treatment) differed according to QoL at FU.	(a) and (b) Multiple linear regression models	(a) QoL at FU differed among the different depression trajectory groups (MCS was significant for all groups: no disorder > remitted disorder > persistent disorder; PCS: no disorder > remitted disorder; remitted disorder < persistent disorder). (b) QoL at FU differed among the different anxiety trajectory groups (MCS was significant for all groups: no disorder > remitted disorder > persistent disorder; PCS: no disorder > persistent disorder, remitted disorder > persistent disorder).
Shigemoto (2020) [72]	PTSD symptoms (PCL-C); Q-LES-Q (psychosocial and physical)	Whether previous PTSD symptoms are associated with QoL at FU.	Longitudinal structural equation model	Previous PTSD symptoms were associated with physical QoL at FU1, but not FU2 or psychosocial QoL at both FUs.
Siqveland (2015) [73]	Depressive symptoms (according to the depression scale from the GHQ-28); PTSD symptoms (PCL-S); WHOQOL-Bref (global and hrqol)	(a) Whether depressive symptoms at BL are associated with QoL at FU. (b) Whether PTSD symptoms at BL are associated with QoL at FU.	(a) and (b) Multiple mixed effects regression analyses	(a) Higher depressive symptoms at BL were associated with reduced QoL at FU. (b) PTSD levels at BL were not significantly associated with reduced QoL at FU.
Spijker (2004) [74]	Depression status (CIDI); Comorbid anxiety (CIDI); SF-36 (social, role emotional)	(a) Whether depression status over time (non-depressed, recovered or depressed (including persistent, relapsing course)) is associated with QoL at FU. Whether comorbid anxiety is associated with QoL at FU (b) in a group with persistent depression and (c) in a group recovered from depression.	ANOVA	(a) QoL at FU was significantly reduced in depressed samples compared to the non-depressed group, and lower in the persistently depressed compared to the recovered group (significant for: role emotional and social). Among the depressed subgroups, there was no significant difference between a persistent or a relapsing course regarding QoL at FU. (b) In the persistently depressed group, comorbid anxiety was significantly associated with reduced QoL at FU (significant for role emotional and social). (c) In those who recovered from depression, comorbid anxiety was significantly associated with reduced QoL (significant for role emotional).
Stegenga (2012) [75]	MDD status according to CIDI (remitted, intermittent and chronic); SF-12 (PCS and MCS)	Whether MDD course (remitted, intermittent and chronic) is associated with QoL over time.	Random coefficient analysis	While change in QoL over time did not differ between course groups, QoL at BL (MCS) was lower in those with a chronic course compared to those who remitted from BL.
Stegenga (2012) [76]	MDD (CIDI); anxiety syndromes (panic disorder and others, PHQ); SF-12 (PCS)	(a) Whether MDD at BL predicts change in QoL over time. (b) Whether anxiety syndrome at BL (compared to no psychiatric diagnosis) predict changes in QoL over time. (c) Whether comorbid anxiety and MDD at BL (compared to no psychiatric diagnosis) predict changes in QoL over time.	(a)–(c) Random coefficient model	(a) While changes in QoL over time did not differ significantly between those with MDD at BL and those without any psychiatric diagnosis, QoL at BL was lower in those with depression. (b) While changes in QoL over time did not differ significantly between those with anxiety syndrome at BL and those without any psychiatric diagnosis, QoL at BL was lower in those with anxiety compared to those without any psychiatric diagnosis. (c) While changes in QoL over time did not differ significantly between those with comorbid anxiety and MDD at BL and those without any psychiatric diagnosis, QoL at BL was lower in those with comorbid anxiety and MDD compared to those without any psychiatric diagnosis.
Stevens (2020) [77]	Posttraumatic stress symptoms (VETR-PTSD); SF-36 (MCS, PCS, physical functioning, bodily pain, general health, role physical, role emotional, mental health, vitality and social functioning)	Whether PTSS at BL is associated with QoL at FU.	Generalized estimating equations	Higher BL PTSS was significantly associated with lower QoL (PCS and MCS) at FU. Using a Bonferroni-corrected alpha value, only the domains of mental health, vitality and social functioning at FU were significantly associated with BL PTSS symptoms. The interaction between time and PTSS at BL was not significant, indicating that PTSS had the same effect on QoL outcomes at both FUs.
Tsai (2007) [78]	Increased post-traumatic stress symptoms (DRPST); MOS SF-36 (physical functioning, role physical, pain, general health, vitality, social functioning, role emotional, mental health, PCS and MCS)	(a) Whether different PTSS trajectory groups over time (persistent PTSS, recovered, delayed and persistently healthy) differed in QoL at FU. (b) Whether increased post-traumatic stress symptoms at BL predicted QoL at FU.	(a) ANOVA (b) Multiple regression models	(a) At FU, those who were persistently healthy had the highest QoL scores (significantly higher compared to the persistent group in all domains; significantly higher than the recovered group for: pain, general health, vitality, mental health and MCS; significantly higher compared to delayed PTSS in all domains). In addition, those with delayed PTSS (significantly lower than the recovered group in all domains except physical functioning) and those with persistent PTSS (significantly lower than recovered group in all domains) had the lowest QoL overall. (b) Increased PTSS at BL was not significantly associated with QoL at FU.
Vulser (2018) [79]	Depressive symptom levels (CES-D score), depression status (CES-D ≥ 19); SF-12v2 (role emotional and social)	Whether depressive symptoms or depression status at BL are associated with QoL at FU.	Generalized linear models	Both the level of depressive symptoms at BL as well as depression status at BL were associated with QoL at FU (significant for: role emotional and social).
Wang (2000) [80]	Depressive symptoms (SCL-90 subscale); anxiety symptoms (SCL-90 subscale); WHOQOL-Bref (total)	(a) Whether depressive symptoms at BL were associated with QoL at FU. (b) Whether anxiety symptoms at BL were associated with QoL at FU.	(a) and (b) Stepwise regression	(a) Higher depressive symptoms at BL were associated with reduced QoL at FU. (b) Anxiety symptoms BL were not included in the final stepwise regression model.
Wang (2017) [81]	Depressive disorder course groups (CIDI); anxiety disorder course (CIDI); SF-36 (MCS, PCS)	(a) Whether QoL at FU differs between three different course groups of depressive disorders (1. no disorder at BL and no suicide attempt until FU; 2. remitted without treatment; 3. persistent disorder/treatment/developed psychiatric co-morbidity/suicide attempt until FU). (b) Whether QoL at FU differs between three different course groups of anxiety disorders (1. no disorder at BL and no suicide attempt until FU; 2. remitted without treatment; 3. persistent disorder/treatment/developed psychiatric co-morbidity/suicide attempt until FU).	(a) and (b) Multiple linear regression	(a) Those with depression at BL that remitted without treatment had lower QoL at FU (significant for MCS and PCS) than those without the disorder and higher QoL at FU (significant for MCS) than those with a persistent disorder. (b) Those with anxiety at BL that remitted without treatment over time had lower QoL at FU than those without the disorder and higher QoL (MCS, but not PCS) than those with a persistent disorder.
Wu (2015) [82]	Depressive symptoms according to CDI; social anxiety symptoms (SASC); QOLS	(a) Whether depressive symptoms at BL are associated with QoL at FU. (b) Whether social anxiety symptoms at BL are associated with QoL at FU.	(a) and (b) Multivariate stepwise forward regression	(a) Higher depressive symptoms at BL were significantly associated with reduced QoL at FU. (b) Higher social anxiety symptoms at BL were not significantly associated with QoL at FU.

Abbreviations: QoL = quality of life; MD = major depression; FU = follow-up; DSM = Diagnostic and Statistical Manual of Mental Disorders; HDRS = Hamilton Depression Rating Scale; PCS = Physical Component Score; MDS = Mental Component Score; MDD = major depressive disorder; ANOVA = analysis of variance; BL = baseline; MDE = major depressive episode; CIDI = Composite International Diagnostic Interview; SF-36 = Short Form 36; AUDADIS = Alcohol Use Disorders and Associated Disabilities Interview Schedule; SF-12 = Short Form 12; PHQ = Patient Health Questionnaire; SF-12v2: Short Form 12, Version 2; HRSD = Hamilton Rating Scale for Depression; HADS = Hospital Anxiety and Depression Scale; QLDS = Quality of Life in Depression Scale; EQ-VAS = EQ Visual Analogue Scale; DIS = Diagnostic Interview Schedule; BDI = Beck Depression Inventory; SCID = Short Children’s Depression Inventory; MINI = Mini-International Neuropsychiatric Interview; PTSD = post-traumatic stress disorder; hrqol = health-related quality of life, IES-15 = Impact of Event Scale 15; Q-DIS = Quick Version of the Mental Health’s Diagnostic Interview Schedule; MADRS = Montgomery–Åsberg Depression Rating Scale; FDD-DSM-IV = Fragebogen zur Depressionsdiagnostik nach Diagnostic and Statistical Manual of Mental Disorders IV; SCAN = Schedule for Clinical Assessment in Neuropsychiatry; DASS = Depression Anxiety Stress Scales; MOS SF = Medical Outcomes Study Short Form; CES-D = Center for Epidemiological Studies Depression Scale; WHOQOL-Bref-TW = WHOQOL-Bref Taiwan Version; MHI-5 = Mental Health Inventory 5; OCD = obsessive compulsive disorder; Y-BOCS = Yale–Brown Obsessive Compulsive Scale; BAI = Beck Angst Inventar; DD = depressive disorder; PD = psychiatric disorder; SAD = social anxiety disorder; Q-LES-Q = Quality of Life Enjoyment and Satisfaction Questionnaire; GHQ-28 = General Health Questionnaire 28; PCL-S = Post-traumatic Stress Disorder Checklist Scale; VETR-PTSD = Vietnam Era Twin Registry Posttraumatic Stress Disorder; DRPST = Disaster-Related Psychological Screening Test; SCL-90 = Symptomcheckliste bei psychischen Störungen 90; SASC = SpLD Assessment Standards Committee; QOLS = Quality of Life Scale; CDI = Children’s Depression Inventory.

**Table 3:** Studies on QoL as the independent variable and depression/anxiety as outcome.

First Author (Year)	Disorder or Symptoms Analyzed; QoL Domains Analyzed	Research Question	Methods	Results
Chou (2011) [83]	Depressive sympt oms (CES-D-20 score); WHOQOL-Bref (total)	Whether QoL at BL is associated with depressive symptoms at FU.	Multiple regression	Lower QoL at BL was associated with higher depressive symptoms at FU.
De Almeida Fleck (2005) [84]	Depression status (remission vs. no complete remission, CIDI and CES-D-20 cutoff >16); QLDS, WHOQOL-Bref (physical, psychological, social and environment), SF-12 (PCS, MCS)	Whether QoL at BL is associated with course of depression (complete remission vs. non-complete remission) in a depressed sample.	Stepwise multiple logistic regression	Disease-specific QoL measure at BL significantly predicted the remission of depression at FU (significant for QLDS).
Hajek (2015) [21]	Depressive symptoms (GDS); EQ-VAS	Whether an initial change in QoL is associated with subsequent changes in depressive symptoms.	Vector autoregressive model	Initial changes in QoL were associated with a subsequent reduction in depression score (significant for total sample and women).
Hoertel (2017) [19]	MD (according to AUDADIS-IV): SF-12v2 (PCS and MCS)	Whether QoL at BL predicted recurrence (vs. remission) or persistence (vs. remission) of MD over time.	Structural equation model	Lower QoL at BL was a predictor of risk of persistence (PCS and MCS) and recurrence of MDE over time.
Johansen (2007) [57]	PTSD symptoms according to IES-15; WHOQOL-Bref (total)	Whether QoL predicted PTSD symptoms at FU.	Structural equation model	QoL did not significantly predict PTSD symptoms at FU.
Kuehner (2009) [20]	Depressive symptoms (MADRS); WHOQOL (overall, physical, psychological, social and environmental)	Whether the lag of levels of QoL predicts future levels of depressive symptoms and whether the association differs by group (formerly depressed inpatients vs. community controls)	Time-lagged linear models	Lower levels of QoL were associated with higher future depressive symptoms (significant for physical, psychological, environmental and overall). The association was not moderated by group status.
Stegenga (2012) [76]	MDD (CIDI); anxiety syndromes (panic disorder and others, PHQ); SF-12 (PCS)	(a) Whether “dysfunction” (i.e., reduced QoL) at BL (mildly reduced, moderately reduced or severely reduced; compared to no reduced QoL) predicts MDD onset over time. (b) Whether “dysfunction” (i.e., reduced QoL) at BL (mildly reduced, moderately reduced or severely reduced; compared to no reduced QoL) predicts anxiety syndrome onset over time. (c) Whether “dysfunction” (i.e., reduced QoL) at BL (mildly reduced, moderately reduced or severely reduced; compared to no reduced QoL) predicts onset of comorbid anxiety and MDD over time.	(a)–(c) Multinomial logistic regressions	(a) Dysfunction (i.e., reduced QoL) at BL was associated with higher odds of onset of MDD over time in the sample of people without a diagnosis at BL (significant for severely reduced QoL). (b) Dysfunction (i.e., reduced QoL) at BL was associated with higher odds of onset of anxiety syndrome over time in the sample of people without a diagnosis at BL (significant for moderately and severely reduced QoL). (c) Dysfunction (i.e., reduced QoL) at BL was associated with higher odds of onset of comorbid anxiety and depression over time in the sample of people without a diagnosis at BL (significant for mild, moderately and severely reduced QoL).
Wu (2016) [85]	Elevated social anxiety symptoms (SASC cutoff 9); QOLS	Whether QoL is associated with changes in elevated social anxiety symptoms over time.	Generalized Estimating Equation	Higher QoL was associated with a decreased risk for developing elevated social anxiety symptoms over time.
Wu (2017) [86]	Elevated depressive symptoms (according to CDI ≥19); QOLS	Whether QoL at BL is associated with elevated depressive symptoms at FU.	Multiple stepwise logistic regression	QoL at BL was not significantly related to depressive symptoms at FU.

Abbreviations: CES-D-20 = Center for Epidemiological Studies Depression Scale 20; BL = baseline; FU = follow-up; QoL = quality of life; CIDI = Composite International Diagnostic Interview; QLDS = Quality of Life in Depression Scale; SF-12 = Short Form 12; PCS = Physical Component Score; MCS = Mental Component Score; GDS = Geriatric Depression Scale; EQ-VAS = EQ Visual Analogue Scale; MD = mental disorder; AUDADIS-IV = Alcohol Use Disorders and Associated Disabilities Interview Schedule; SF-12v2 = Short Form 12 Version 2; PTSD = post-traumatic stress disorder; IES-15 = Impact of Event Scale 15; MADRS = Montgomery–Åsberg Depression Rating Scale; MDD = major depressive disorder; PHQ = Patient Health Questionnaire; SASC = SpLD Assessment Standards Committee; QOLS = Quality of Life Scale; CDI = Children’s Depression Inventory.

## Data Availability

Not applicable.

## Supplementary Materials

The following are available online at https://www.mdpi.com/article/10.3390/ijerph182212022/s1, Table S1: detailed descriptive information for included studies (*n* = 47); Figure S1: forest plot for differences in SF MCS at FU among adults with and without depressive disorders at BL; Figure S2: forest plot for differences in SF PCS at FU among adults with and without depressive disorders at BL; Figure S3: forest plot for differences in SF MCS at FU among adults with and without depressive disorders at BL (sensitivity analysis); Figure S4: forest plot for differences in SF PCS at FU among adults with and without depressive disorders at BL (sensitivity analysis); Figure S5: forest plot for BL differences in SF MCS among adults with MD at BL with and without remitting courses over time; Figure S6: forest plot for BL differences in SF PCS among adults with MD at BL with and without remitting courses over time; Figure S7: forest plot for FU differences in SF MCS among adults with depressive disorders at BL with and without remitting courses; Figure S8: forest plot for FU differences in SF PCS among adults with depressive disorders at BL with and without remitting courses; Figure S9: forest plot for FU differences in SF MCS among adults with anxiety disorders at BL with and without remitting courses; Figure S10: forest plot for FU differences in SF PCS among adults with anxiety disorders at BL with and without remitting courses.
