# Supplementary material for: Anxiety, Depression and Quality of Life—A Systematic Review of Evidence from Longitudinal Observational Studies
Source: Int J Environ Res Public Health. 2021 Nov 16;18(22):12022. doi: 10.3390/ijerph182212022 (PMC8621394; doi:10.3390/ijerph182212022)
Supplement: Supplementary file 1 [file ijerph-18-12022-s001.zip › ijerph-1443304-supplementary.pdf]

Appendix. Descriptive table and forest plots for meta-analyses of SF MCS and PCS scores

Table S1. Detailed descriptive information for included studies (N = 47)

First author (year)	Title of the paper	Country	Sample description; recruitment setting	Sample characteristics at BL			Assessment of anxiety/ depression and prevalence percentage /mean (SD) scores at BL	Assessment of QoL and mean (SD) scores at BL	Length of follow-up(s)	Quality assessment rating
				N	Age: mean (SD), range	% Female				
Ardal (2013)	Health-related quality of life in recurrent major depressive disorder-a 10-year follow-up study	Norway	Prospective study of patients with recurrent MD and who were in recovery at FU, recruited from psychiatric clinics and institutions, and matched healthy control group; Other (health care and healthy controls)	$N_{analytical\ sample}$ = 28 (14 MD patients and 14 controls)	50.3 (9.7)	Not reported	DSM-IV criteria for recurrent MD, HDRS<br><br>HDRS scores in MD-group at BL: 21.6 (3.6)	SF-36<br><br>SF-36 scores at B<br>• Physical functioning: 78.3 (22.3)<br>• Physical role: 54.5 (48.6)<br>• Pain: 70.9 (28.9)<br>• General health: 64.2 (28.8)<br>• Vitality: 40.7 (26.0)<br>• Social functioning: 61.0 (40.7)<br>• Emotional role:52.4 (46.8)<br>• Psychological health: 56.2 (29.9)<br>• PCS: 61.7 (26.7)<br>• MCS: 54.8 (31.9)<br>• Total: 59.8 (29.9)	10 years (1 FU)	Poor
Buist-Bouwman (2004)	Functioning after a major depressive	Netherlands	Prospective study of adults from the Dutch general	N_{BL} = 7,076; N_{FU} =	Total: 41.4,	Total: 51.7, MDE	CIDI (DSM-III-R) for MDE	SF-36	3 years (2 FUs, one at 1 year	Good

Appendix. Descriptive table and forest plots for meta-analyses of SF MCS and PCS scores

	episode: complete or incomplete recovery?	population (NEMESIS), Subgroup of adults who recovered from a new MDE; General population	4,796; $N_{MDE\ subsample}$ = 165	MDE subsampl e: 38.7, NEMESI S: 18-64	subsampl e: 69%	Incidence and remission of MDE-group in total sample: 3.8%	SF-36 scores at BL for total sample<br>• physical functioning: 92.4 (20.4)<br>• physical role: 86.9 (28.8)<br>• vitality: 73.0 (17.1)<br>• pain: 85.9 (20.7)<br>• psychological health: 83.5 (13.0)<br>• psychological role: 94.6 (19.3)<br>• social functioning: 91.0 (19.3)<br>• general health: 75.2 (16.9)<br><br>SF-36 scores at BL for MDE subsample<br>• physical functioning: 88.0 (17.0)<br>• physical role: 78.2 (35.2)<br>• vitality: 63.3 (18.1)<br>• pain: 80.1 (23.5)<br>• psychological health: 73.9 (16.5)<br>• psychological role: 85.7 (30.4)	and one at 3 years after BL)

Appendix. Descriptive table and forest plots for meta-analyses of SF MCS and PCS scores

								• social functioning: 82.8 (19.7)<br>• general health: 68.3 (18.9)		
Cabello (2014)	Risk factors for incidence and persistence of disability in chronic major depression and alcohol use disorders: longitudinal analyses of a population-based study	United States of America	Representative sample of non-institutionalized U.S. general population aged 18 years and older (NESARC), Subgroup of adults with chronic MD; General population	N_{BL} = 43,093; N_{FU} = 34,653; $N_{chronic\ MD\ subgroup}$ = 272	NESARC: 46.0 (17.4); 18-98	NESARC: 58.0%	AUDADIS interview (DSM-IV) for MD and severity of MD symptoms<br><br>Chronic MD in sample: 0.008%	SF-12<br><br>SF-12 scores for NESARC<br>• physical functioning: 34.5 (10.1)<br>• physical role: 50.9 (10.3)<br>• vitality: 53.9 (10.8)<br>• pain: 49.9 (12.2)<br>• mental health: 51.9 (10.5)<br>• emotional role: 50.7 (10.2)<br>• social functioning: 51.7 (9.9)<br>• general health: 50.1 (12.2)	3 years (1 FU)	Good
Cerne (2013)	Quality of life in patients with depression, panic syndrome, other anxiety syndrome, alcoholism and chronic somatic diseases: a longitudinal	Slovenia	Adult patients recruited in family practices regardless of their reason for visit; Health care	N_{BL} = 1,121; N_{FU2} = 714; $N_{analytical\ sample}$ = 516	48.0 (14.6); 18-75	62.8%	CIDI for depression (DSM-IV criteria), PHQ modules for panic syndrome and other anxiety syndromes<br><br>Disorders at BL:<br>• Depression: 15.3%	SF-12<br><br>SF-12 scores for analytical sample<br>• MCS: 51.6 (8.2)<br>• PCS: 44.9 (9.0)	2 years (2 FUs, one at 6 and one at 24 months after BL)	Fair

	study in Slovenian primary care patients						• Panic syndrome: 6.4%<br>• Other anxiety syndrome: 2.5%			
Chin (2015)	12-Month naturalistic outcomes of depressive disorders in Hong Kong's primary care	China/Hong Kong	Adults recruited in primary care who screened positive for depression; Health care	N_{BL} = 10,179; $N_{FU\ cohort}$ = 4,358; N_{FU3} = 3,505; $N_{depression\ subgroup}$ = 539	49.0 (17.1); depression subgroup: 46.9 (17.4); 18-94	56.6%; depression subgroup: 68.9%	PHQ-9, CES-D-20<br><br>Depression screened positive at BL among FU cohort: 12.4%	SF-12v2<br><br>SF-12v2 scores for depression subgroup at BL<br>• PCS: 42.7 (11.9)<br>• MCS: 37.8 (12.6)	1 year (3 FUs; one at 3, 6, and 12 months after BL)	Fair
Chou (2011)	Interaction between pre- and post-migration factors on depressive symptoms in new migrants to Hong Kong from Mainland China	China/Hong Kong	Adults newly migrating to Hong Kong from mainland China; Other (migration sample)	N_{BL} = 449; N_{FU} = 347	34.1 (8.7), 18-67	87.3%	CES-D-20<br><br>CES-D-20 at BL: 11.4 (10.0)	WHOQOL-Bref<br><br>WHOQOL-Bref at BL: 99.3 (13.3)	1 year (1 FU)	Good
Chung (2012)	Quality of life in major depressive disorder: the role of pain and pain catastrophizing cognition	China	Adult outpatients or inpatients with new or first MDD episode; Health care	N_{BL} = 91; N_{FU} = 82	48.3 (9.5), 20-62	80.2%	DSM-IV, HADS, $HRSD_{17}$<br><br>HADS-anxiety at BL: 11.8 (4.3)<br><br>HADS-depression at BL: 11.6 (4.5)<br><br>$HRSD_{17}$ at BL: 24.3 (4.8)	SF-36<br><br>SF-36 at BL<br>• physical: 72.7 (18.7)<br>• physical-role: 20.7 (32.6)<br>• pain: 38.2 (24.3)<br>• general health: 28.2 (20.0)<br>• vitality: 29.3 (19.1)<br>• social: 38.7 (28.3)	3 months (1 FU)	Fair

Appendix. Descriptive table and forest plots for meta-analyses of SF MCS and PCS scores

								• emotional: 13.8 (27.7)<br>• mental health: 35.3 (18.9)<br>• PCS: 33.4 (10.8)<br>• MCS: 25.1 (11.3)		
De Almeida Fleck (2005)	Major depression and its correlates in primary care settings in six countries. 9-month follow-up study	Cross-national (Australia, Brazil, Israel, Spain, Russia, United States of America)	Prospective study of adult primary care patients with new and/or untreated MD at BL (LIDO study); Health care	$N_{analytical\ sample}$ = 968	40.8 (14.6), 18-75	71.3%	CES-D-20, CIDI<br><br>CES-D-20 at BL: 29.1 (10.6)	QLDS, WHOQOL-Bref, SF-12<br><br>QLDS at BL: 12.0 (7.6)<br><br>WHOQOL-Bref at BL<br>• physical: 12.1 (2.8)<br>• psychological: 11.4 (2.6)<br>• social: 11.9 (3.3)<br>• environment: 12.3 (2.4)<br><br>SF-12 at BL<br>• PCS: 41.7 (11.3)<br>• MCS: 34.0 (9.8)	9 months (1 FU)	Fair
Diehr (2006)	Synchrony of change in depressive symptoms, health status, and quality of life in persons with clinical depression	Cross-national (Australia, Brazil, Israel, Spain, Russia, United States of America)	Prospective study of adult primary care patients with untreated MD at BL (LIDO study); Health care	N_{BL} = 1,180; N_{FU} = 982	40.8 (14.6), 17-76	71.0%	CES-D, CIDI<br><br>CES-D at BL: 29.0	SF-12, QLDS, WHOQOL-Bref<br><br>SF-12 at BL<br>• MCS : 34.1<br>• PCS: 42.0<br><br>QLDS at BL: 11.9<br><br>WHOQOL-Bref at BL:<br>• Environment: 12.3	9 months (3 FUs, one at 6 weeks, 3 months, and 9 months after BL)	Fair

								• physical: 12.2<br>• psychological: 11.4<br>• social: 12.0<br>• social: 12.0		
Hajek (2015)	Complex coevolution of depression and health-related quality of life in old age	Germany	Older adults from a representative, population-based cohort recruited in primary care (AgeCoDe study); Health care	N_{BL} = 3,287; $N_{analytical\ sample}$ = 2,696	82.6 (3.4), 75+	66.0%	GDS<br><br>GDS at BL: 2.5 (2.5)	EQ-VAS<br><br>EQ-VAS at BL: 65.4 (17.9)	6 years (4 FUs, each 18 months apart)	Good
Hasche (2010)	Quality of Life Outcomes for Depressed and Non-Depressed Older Adults in Community Long Term Care	United States of America	Older adults recruited as new clients of a community long-term care program; Health care	N_{BL} = 615; $N_{analytical\ sample}$ = 551	72.4 (7.9); 60+	77.1%	DIS, CES-D<br><br>Depression at BL according to screener: 25.6%	SF-8<br><br>SF-8 at BL<br>• PCS-8: 33.2 (11.1)<br>• MCS-8: 36.3 (20.3)	1 year (2 FUs, one at 6 and one at 12 months after BL)	Fair
Heo (2008)	A prospective study on changes in health status following flood disaster	Korea	Community-recruited sample from agricultural population experiencing flood disaster; Other (agricultural population experiencing flood disaster)	N_{BL} = 83; N_{FU} = 67; $N_{analytical\ sample}$ = 58	53.6 (14.6)	51.7%	BDI<br><br>Descriptive information for BL BDI not available (only included at FU assessment)	SF-36-K<br><br>SF-36-K total score at BL: 59.6 (19.8)	1.5 years (1 FU)	Fair
Ho (2014)	Coexisting medical comorbidity and depression: multiplicative effects on health outcomes in older adults	Singapore	Older adults recruited from the community (SLAS); General population	N_{BL} = 2,805; N_{FU} = 1,850; $N_{analytical\ sample}$ = 1,788	66.0 (7.4), 55+	65.3%	GDS<br><br>Depression at BL: 11.4%<br><br>GDS at BL: 1.8 (2.6)	SF-12<br><br>SF-12 at BL<br>• PCS: 48.6 (7.2)<br>• MCS: 54.2 (7.9)	2 years (1 FU)	Fair

Appendix. Descriptive table and forest plots for meta-analyses of SF MCS and PCS scores

Hoertel (2017)	A comprehensive model of predictors of persistence and recurrence in adults with major depression: Results from a national 3-year prospective study	United States of America	Representative sample of civilian, noninstitutionalized, adult US population (NESARC), respondents with MDE in past year at BL; General population	N_{FU} = 34,653; $N_{analytical\ sample\ with\ past\text{-}year\ MDE\ at\ BL}$ = 2,587	39.5 (1.0)	66.6%	DSM-IV, AUDADIS-IV<br><br>Past-year MDE at BL: 7.5%	SF-12v2<br><br>SF-12v2<br>• PCS: 47.9 (1.5)<br>• MCS: 43.6 (2.7)	3 years (1 FU)	Good
Hussain (2016)	The relationship between psychiatric morbidity and quality of life: interview study of Norwegian tsunami survivors 2 and 6 years post-disaster	Norway	Sample of adult Norwegian tourists who have survived a Tsunami; Other (tourists who survived a tsunami)	N_{BL} = 62; N_{FU} = 58	40.6 (11.3)	46.8%	SCID, MINI<br><br>Disorders at BL:<br>• PTSD: 11.3%<br>• Specific phobia: 29.0%<br>• Other anxiety disorders: 27.4%<br>• Depressive disorders: 22.6%	WHOQOL-Bref (full questionnaire at BL, reduced number of items at FU)<br><br>WHOQOL-Bref at BL:<br>• general QoL: 4.0 (0.9)<br>• hrqol: 3.6 (1.2)	4 years (1 FU)	Poor
Joffe (2012)	Lifetime history of depression and anxiety disorders as a predictor of quality of life in midlife women in the absence of current illness episodes	United States of America	Midlife women without current mental illness episode (SWAN study); General population	N_{BL} = 443; $N_{analytical\ sample}$ = 425	45.6 (2.5)	100%	SCID<br><br>Lifetime history of disorder at FU:<br>• anxiety or depression: 51.8%<br>• depression only: 38.1%<br>• anxiety only: 4.9%<br>• comorbid	SF-36<br><br>SF-36 at BL not reported	6 years (6 FUs, one each year)	Fair

Appendix. Descriptive table and forest plots for meta-analyses of SF MCS and PCS scores

							depression and anxiety: 22.8%			
Johansen (2007)	The predictive value of post-traumatic stress disorder symptoms for quality of life: a longitudinal study of physically injured victims of non-domestic violence	Norway	Non-domestic violence victims who had sought assistance from emergency department or filed a police report; Several settings: health care and non-domestic violence victims filing police report	N_{BL} = 173; $N_{analytical\ sample}$ = 70	33 (12.3), 18-75	17%	IES-15<br><br>IES-15 at BL:<br>• Total: 26.0 (18.4)<br>• Intrusion: 13.6 (10.1)<br>• Avoidance: 12.3 (10.5)	WHOQOL-Bref<br><br>WHOQOL-Bref at BL:<br>• Physical health: 14.9 (3.6)<br>• Psychological: 13.9 (3.1)<br>• Social: 14.7 (3.2)<br>• Environmental: 14.5 (2.7)<br>• Overall health: 3.6 (1.0)<br>• Overall QoL: 3.4 (1.1)	12 months (2 FUs)	Fair
Kramer (2003)	Service utilization and outcomes in medically ill veterans with posttraumatic stress and depressive disorders	United States of America	Male veterans hospitalized for somatic illnesses with and without mental disorders; Health care	N_{BL} = 743; N_{FU2} = 582	18+	0%	Q-DIS, SCL-90<br><br>Current or lifetime disorders at BL:<br>• depression: 4.8%<br>• PTSD: 6.2%<br>• comorbid depression & PTSD: 6.2%	SF-36<br><br>SF-36 adjusted means<br>• physical: 45.1 (3.9)<br>• physical-role: 31.4 (5.8)<br>• pain: 50.3 (6.3)<br>• general health: 46.6 (4.9)<br>• energy/fatigue: 42.0 (6.1)<br>• social: 64.4 (6.3)<br>• emotional: 75.2 (13.1)	1 year (2 FUs, one at 3, and 12 months after BL)	Fair

Appendix. Descriptive table and forest plots for meta-analyses of SF MCS and PCS scores

								• mental health: 73.6 (8.6)		
Kuehner (2009)	Subjective quality of life aspects predict depressive symptoms over time: results from a three-wave longitudinal study	Germany	Formerly depressed inpatients, recruited at discharge from psychiatric hospital and matched community controls; several settings (health care and general population)	N_{BL} = 182; $N_{analytical\ sample}$ = 158 (among those n = 82 depressed patients; n = 76 communit y controls with follow-up data)	45.0 (12.4), 18-70	49.4%	ICD-10 diagnosis (F32, F33, F34.1) at hospital stay; SCID for MD (DSM-IV), MADRS<br><br>In depression subgroup: 78% fully remitted, 11% partly remitted, 11% non-remitted at BL<br><br>In community group: 2.6% minor depression at BL	WHOQOL-Bref<br><br>WHOQOL-Bref at BL not reported	3.5 years (2 FUs, one at 5, and 41 months after BL; BL assessment was one month after hospital discharge)	Fair
Kuehner (2012)	Response styles to depressed mood affect the long-term course of psychosocial functioning in depressed patients	Germany	Formerly depressed inpatients, recruited at discharge from psychiatric hospital; Health care	N_{BL} = 91; $N_{analytical\ sample}$ = 71	45.1 (12.7), 19-70	53.5%	ICD-10 diagnosis at hospital stay; SCID for MD, FDD-DSM-IV, MADRS<br><br>At BL: 19.7% with first MDE, 58.5% with at least third MDE	WHOQOL-Bref<br><br>WHOQOL-Bref at BL not reported	5.5 years (3 FUs, one at 5, 41, and 65 months after BL; BL assessment was one month after hospital discharge)	Fair
Lenert (2000)	Estimation of utilities for the effects of depression from the SF-12	United States of America	Primary care sample of patients with current MDD or dysthymia (Course of Depression study); Health care	N_{BL} = 395; N_{FU} = 295; $N_{analytical\ sample}$ = 241	43.7	73%	DSM-III diagnosis (DIS)<br><br>MDD: 40%	SF-12<br><br>SF-12 at BL:<br>• PCS: 44.5<br>• MCS: 39.0	2 years (2 FUs)	Fair
Mars (2015)	Longitudinal symptom course in adults with	Wales	Recurrently depressed parents (Early Prediction of Adolescent	N_{BL} = 337; $N_{analytical}$	42.2 (5.4)	91.8%	SCAN interview and clinical review by psychiatrist	EQ-5D-3L (without anxiety/depression item)	Mean time between assessment s: BL-	Fair

	recurrent depression: Impact on impairment and risk of psychopathology in offspring		Depression study); several settings (primary care, previous sample, community)	sample = 233			MDD at BL: 22.4%<br><br>SCAN number of depressive symptoms at BL: 2.4 (2.6)	EQ-5D-3L at BL not reported	FU1: 16.2 months, FU1-FU2: 12.5 months (2 FUs)	
Moutinho (2019)	Mental health and quality of life of Brazilian medical students: Incidence, prevalence, and associated factors within two years of follow-up	Brazil	Medical students from Brazil, recruited in medical school; Other (medical school)	N_{BL} = 743; $N_{analytical\ sample}$ = 312	21.0 (2.6)	64.1%	DASS<br><br>DASS Depression scale at BL: 4.1 (3.8)<br><br>Depression (DASS Depression scale cutoff 9) at BL: 10.5%<br><br>DASS Anxiety scale at BL: 3.5 (3.3)<br><br>Anxiety (DASS Anxiety scale cutoff 7) at BL: 12.8%	WHOQOL-Bref<br><br>WHOQOL-Bref at BL:<br>• physical: 3.5 (0.5)<br>• psychological: 3.0 (0.5)<br>• social: 3.8 (0.6)<br>• environmental: 3.5 (0.5)	2 years (4 FUs, in the middle of the semester)	Fair
Ormel (1999)	Onset of disability in depressed and non-depressed primary care patients	Cross-national (Turkey, India, Germany, Netherlands, Nigeria, United Kingdom, France, United States of America, China, Italy)	Adult primary care attendees without disability (i.e. reduced QoL) at BL; WHO Collaborative Study on Psychological Problems in General Health Care; Health care	N_{BL} = 3,839; N_{FU2} = 2,089; $N_{analytical\ sample}$ = 1,051	15-65	not reported	CIDI (ICD-10 depression, F32/F33)<br><br>Depression at BL: 14%	MOS SF 6-item Physical functioning scale<br><br>MOS SF 6-item Physical functioning at BL: 3.1 (3.3)	1 year (2 FUs, one at 3 and one at 12 months after BL)	Fair
Pakpour (2018)	Predictors of oral health-related quality of life in Iranian	Iran	Adolescents from Iran, recruited in school; School	N_{BL} = 1,529; $N_{analytical\ sample}$ = 1,052	15.1 (2.1)	54.6%	MDAS<br><br>MDAS at BL: 14.7 (5.4)	PedsQL 4.0<br><br>PedsQL 4.0 at BL:<br>• general: 65.9 (18.7)	18 months (1 FU)	Good

	adolescents: A prospective study							• physical: 74.5 (25.5)<br>• emotional: 71.1 (24.4)<br>• social: 51.5 (16.1)<br>• school: 60.4 (22.7)		
Pan (2012)	A longitudinal study of the predictors of quality of life in patients with major depressive disorder utilizing a linear mixed effect model	Taiwan	MD patients recruited in outpatient psychiatric clinic; Health care	N_{BL} = 104; $N_{analytical\ sample}$ = 70	49.1 (12.5)	72.1%	ICD-10 diagnosis at BL; CES-D<br><br>CES-D at BL: 37.9 (15.6)	WHOQOL-Bref-TW<br><br>WHOQOL-Bref-TW at BL:<br>• overall: 3.2 (0.9)<br>• physical: 54.5 (16.0)<br>• psychological: 47.6 (19.2)<br>• social: 48.8 (17.1)<br>• environmental: 56.5 (14.4)	On average 26.1 months (1 FU)	Fair
Panagioti (2018)	Effect of health literacy on the quality of life of older patients with long-term conditions: a large cohort study in UK general practice	United Kingdom	Older adults with at least one long-term condition recruited in general practice (CLASSIC study); Health care	N_{BL} = 4,377; N_{FU} = 3,390	74.5 (6.8), 65+	52.6%	MHI-5<br><br>MHI-5 at BL: 67.3 (22.4)	WHOQOL-Bref<br><br>WHOQOL-Bref at BL:<br>• physical: 60.0 (22.3)<br>• psychological: 69.6 (17.7)<br>• environment: 68.4 (20.2)<br>• social relationships: 72.5 (16.4)	1 year (1 FU)	Fair
Pyne (1997)	Preliminary longitudinal assessment of quality of life in patients	United States of America	Patients with MD and a community comparison group without MD; several	$N_{analytical\ sample}$ = 199 (among those 118	52.0 (13.5)	22.1%	SADS or SCID (DSM-III), DIS, HAM-D	QWB<br><br>QWB at BL: 0.7 (0.1)	6 months (1 FU)	Poor

	with major depression		settings (health care and general population)	patients with MD and 81 communit y controls)			HAM-D at BL: 9.1 (10.0)			
Remmerswaal (2020)	Four-year course of quality of life and obsessive–compulsive disorder	Netherlands	OCD patients referred to mental health care centers (NOCDA study); Health care	$N_{analytical\ sample}$ = 239	37.3 (10.9)	54%	SCID (DSM-IV), Y-BOCS, BDI, BAI<br><br>Chronic OCD: 60.3%<br>Intermittent OCD: 9.2%<br>Remitting OCD: 30.5%<br><br>Y-BOCS: 28.8 (7.1)<br>BAI: 17.4 (11.3)<br>BDI: 15.6 (9.7)	EQ-5D<br><br>EQ-5D at BL: 0.7 (0.3)	4 years (2 FUs, one at 2 and one at 4 years)	Fair
Rhebergen (2010)	Trajectories of recovery of social and physical functioning in major depression, dysthymic disorder and double depression: a 3-year follow-up	Netherlands	Adult general population sample (NEMESIS); General population	N_{BL} = 7,076; $N_{analytical\ sample}$ = 4,381	42.0, 18-64	54.3%	CIDI diagnosis for MDD, Dysthymia or double depression, and comorbid anxiety disorders<br><br>Disorders at BL where remission was achieved at FU:<br>MDD: 1.4%<br>Dysthymia: 0.01%<br>Double depression: 0.01%	SF-36 (physical health summary score)<br><br>SF-36 at BL not reported	3 years (2 FUs, one at 1 year, and one at 3 years after BL)	Good
Rozario (2006)	Changes in the SF-12 among depressed elders six months after discharge from an inpatient geropsychiatric unit	United States of America	Older adults with depression, recruited in inpatient geropsychiatric care and discharged to community setting (Service Use of Depressed	$N_{analytical\ sample}$ = 154	75.7 (7.0)	72%	GDS<br>GDS at time of discharge: 12.2 (6.9)	SF-12 (MCS, PCS)<br><br>SF-12 at BL:<br>• MCS: 41.6 (11.7)<br>• PCS: 30.8 (11.3)	(2 FUs, one at 6 weeks, and one at 6 months after discharge)	Fair

Appendix. Descriptive table and forest plots for meta-analyses of SF MCS and PCS scores

			Elders after Acute Care Study); Health care							
Rubio (2014)	Effect of first episode axis I disorders on quality of life	United States of America	Representative sample of civilian, noninstitutionalized, adult US population (NESARC); General population	N_{BL} = 43,093; $N_{analytical\ sample}$= 34,653	18+	not reported	AUDADIS-IV<br><br>Incident mental disorder at FU:<br>• MDD: 4.3%<br>• GAD: 3.6%<br>• SAD: 2.1%<br>• PD: 2.4%<br>• SP: 6.0%	SF-12 (MCS)<br><br>SF-12 MCS at BL: 53.0	3 years (1 FU)	Good
Rubio (2013)	Quality of life following remission of mental disorders: findings from the National Epidemiologic Survey on Alcohol and Related Conditions	United States of America	Representative sample of civilian, noninstitutionalized, adult US population (NESARC); General population	N_{BL} = 43,093; $N_{analytical\ sample}$= 34,653	18+	not reported	AUDADIS-IV<br><br>Remission from mental disorders at FU:<br>• MDD: 3.8%<br>• GAD: 1.6%<br>• SAD: 2.2%<br>• PD: 1.7%<br>• SP: 5.5%	SF-12 (MCS)<br><br>SF-12 MCS at BL not reported for entire sample	3 years (1 FU)	Good
Sareen (2013)	Common mental disorder diagnosis and need for treatment are not the same: findings from a population-based longitudinal survey	United States of America	Representative sample of civilian, noninstitutionalized, adult US population (NESARC); General population	N_{BL} = 43,093; $N_{analytical\ sample}$= 34,653	18+	not reported	AUDADIS-IV<br><br>Past year disorders at BL:<br>• Anxiety disorder: 10.0%<br>• Depressive disorder (dysthymia or depression): 5.9%	SF-12<br><br>SF-12 at BL not reported	3 years (1 FU)	Good

Appendix. Descriptive table and forest plots for meta-analyses of SF MCS and PCS scores

Shigemoto (2020)	Social cohesion and quality of life among survivors of a natural disaster	United States of America	Residents of Galveston or Chambers Counties experiencing Hurricane Ike (Galveston Bay Recovery Study); other (hurricane survivors)	$N_{analytical\ sample}$ = 658	Not reported	Not reported	PCL-C<br><br>PCL-C at BL: 1.6 (0.8)	Q-LES-Q<br><br>Q-LES-Q at BL:<br>• psychosocial: 3.4 (0.9)<br>• physical: 3.8 (0.9)	1 year (2 FUs, one at 6 and 15 months after the disaster)	Good
Siqveland (2001)	Posttraumatic growth, depression and posttraumatic stress in relation to quality of life in tsunami survivors: a longitudinal study	Norway	Sample of Norwegian tourists who have survived a Tsunami; other (tsunami survivors)	N_{BL}= 63; $N_{analytical\ sample}$ = 58	40.5 (11.3)	55.2%	PCL, depression subscale of the GHQ-28<br><br>GHD-depression at BL: 2.7 (4.2)<br><br>PCL at BL: 31.6 (15.1)	WHOQOL-Bref (full questionnaire at BL, reduced number of items at FU)<br><br>WHOQOL-Bref at BL:<br>• general QoL: 4.0 (0.9)<br>• hrqol : 3.7 (1.2)	4 years (1 FU)	Fair
Spijker (2004)	Functional disability and depression in the general population. Results from the Netherlands Mental Health Survey and Incidence Study (NEMESIS)	Netherlands	Cohort with newly originated MDE from adult general population sample (NEMESIS); General population	$N_{analytical\ sample}$ = 250	41.7 (10.7), 18-64	66.8%	CIDI (DSM-III-R)<br><br>MDE: 5.2%, among those 27.6% persistent cases, 72.4% recovered at FU	MOS-SF-36 (role emotional, social)<br><br>MOS-SF-36 at BL:<br>• role emotional: 77.4 (37.1)<br>• social: 78.8 (21.4)	3 years (2 FUs, one at 1 year and one at 3 years after BL)	Good
Stegenga (2012a)	The natural course and outcome of major depressive disorder in primary care: the PREDICT-NL study	Netherlands	Adult primary care cohort recruited irrespective of their reason for consulting (PREDICT-NL); subgroup with depression at BL; Health care	$N_{BL(total\ sample)}$= 1,338; $N_{FU(total\ sample)}$= 759; $N_{BL(depression\ subgroup)}$= 174; $N_{FU\ (depression}$	51 (17)	63%	CIDI (DSM-IV), PHQ-9<br><br>MDD course in sample with depression at BL: 17% chronic, 40% fluctuating course, and 43% remitted	SF-12<br><br>SF-12 at BL:<br>• MCS: 32 (9)<br>• PCS: 47 (11)	3.25 years (3 FUs, one at 6, 12, and 39 months after BL)	Fair

Appendix. Descriptive table and forest plots for meta-analyses of SF MCS and PCS scores

				subgroup)= 100			PHQ-9 at BL: 10 (6)			
Stegenga (2012b)	Depression, anxiety and physical function: exploring the strength of causality	Cross-national (United Kingdom, Spain, Portugal, Slovenia)	Adult primary care cohort (PredictD); Health care	N_{BL} = 4,757; N_{FU2} = 4,006; $N_{analytical\ sample}$ = 3,029	50 (15), 18-75	67%	CIDI (DSM-IV), PHQ anxiety and panic syndrome modules<br><br>MDD only: 6%<br>Anxiety only: 9%<br>Comorbid MDD and anxiety: 7%	SF-12 (PCS)<br><br>SF-12 PCS at BL: 43 (11)	2 years (3 FUs, one at 6, 12, and 24 months after BL)	Good
Stevens (2020)	Predicting health-related quality of life in trauma-exposed male veterans in late midlife: a 20 year longitudinal study	Vietnam	Community-dwelling, non-patient male twins who both served in the military between 1965-75 (VETSA); other setting (military veterans)	$N_{analytical\ sample}$ = 775	37.9 (2.5)	0%	VETR-PTSD<br><br>Not reported	SF-36<br><br>SF-36 at FU1:<br>• bodily pain: 9.0 (2.3)<br>• general health: 18.8 (4.2)<br>• physical functioning: 26.5 (4.0)<br>• role physical: 7.2 (1.4)<br>• role emotional: 5.6 (0.9)<br>• mental health: 24.0 (4.3)<br>• vitality: 16.1 (4.0)<br>• social functioning: 8.8 (1.7)	20 years (2 FUs)	Fair
Tsai (2007)	Three-year follow-up study of the relationship between posttraumatic stress symptoms and quality of life among	Taiwan	Earthquake survivors recruited from an earthquake-affected township; Other (earthquake survivors)	N_{BL} = 4,223; N_{FU} = 1,756	54.5 (16.7), 16-98	54.4%	DRPST<br><br>PTSS: 23.8%	MOS-SF-36<br><br>MOS-SF-36 at BL:<br>• Physical: 84.5<br>• Role physical: 66.9<br>• Social: 86.7	2.5 years (1 FU)	Fair

	earthquake survivors in Yu-Chi, Taiwan							• Mental health: 66.7<br>• Role emotional: 68.6<br>• General health: 58.7<br>• Pain: 80.6<br>• Vitality: 58.1<br>• PCS: 49.2<br>• MCS: 46.6		
Vulser (2018)	Depression, Cognitive Functions, and Impaired Functioning in Middle-Aged Adults From the CONSTANCES Cohort	France	Population-based study of middle-aged adults (CONSTANCES Cohort); General population	$N_{analytical\ sample}$ = 7,426	57.8 (7.2), 45+	52.2%	CES-D<br><br>CES-D at BL: 9.9 (8.4), depressed (CES-D ≥ 19): 13.2%	SF-12v2<br><br>SF-12v2 at FU:<br>• role emotional: 8.5 (1.8)<br>• Social: 4.1 (0.9)	2 years (1 FU)	Good
Wang (2000)	Post-earthquake quality of life and psychological well-being: longitudinal evaluation in a rural community sample in northern China	China	Earthquake survivors recruited from an earthquake-affected township and controls recruited from unaffected village; Several settings (earthquake survivors and controls)	Earthquake survivors: N_{BL} = 335; N_{FU} = 263, and N = 176 controls	39.7 (10.8)	53.4%	SCL-90<br><br>SCL-90 scores at BL:<br>• Anxiety: 1.0 (0.8) [a]<br>• Depression: 0.9 (0.8) [a]	WHOQOL-Bref<br><br>WHOQOL-Bref at BL:<br>• physical: 3.5 (0.7)<br>• psychological: 3.3 (0.7)<br>• environment: 2.9 (0.5)<br>• social relationships: 3.8 (0.6)<br>• total: 3.3 (0.5)	6 months (1 FU)	Fair
Wang (2017)	Common Mental Disorder Diagnosis and Need for Treatment are Not the Same: Findings from	Netherlands	Adult general population sample (NEMESIS); General population	N_{BL} = 7,076; N_{FU} = 5,618	18-64	not reported	CIDI (DSM-III-R)<br><br>12-month disorder at BL:<br>• depression: 6.1%<br>• anxiety: 11.3%	SF-36<br><br>SF-36 at BL not reported	1 year (1 FU)	Good

	the NEMESIS Study									
Wu (2015)	Children in rural China enjoyed a significant increase in quality of life from 2009 to 2011	China	Longitudinal study of children in rural China, recruited in school; School	N_{BL} = 1,047; N_{FU} = 816	11.2 (2.2), 7-16	48.3%	CDI, SASC<br><br>CDI at BL: 11.3 (6.1)<br><br>SASC at BL: 5.6 (3.7)	Quality of life scale<br><br>Quality of life scale at BL: 71.1 (14.6)	2 years (1 FU)	Good
Wu (2016)	The risk and protective factors in the development of childhood social anxiety symptoms among Chinese children	China	Prospective study of Chinese children, recruited in school; School	N_{BL} = 1,047; N_{FU2} = 816	11.2 (2.2)	48.3%	SASC<br><br>Social anxiety at BL: 15.7%<br><br>SASC at BL: 5.6 (3.7)	Quality of life scale<br><br>Quality of life scale at BL: 71.1 (14.5)	2 years (2 FUs, each 12 months apart)	Good
Wu (2017)	A prospective study of psychological resilience and depression among left-behind children in China	China	Prospective study of children's mental health in China, left-behind children, recruited in school; School	$N_{analytical\ sample}$ = 386	12.2 (2.1), 8-17	50.3%	CDI<br><br>Depression at BL: 12.7%<br><br>Mean (SD) at BL: 11.5 (6.3)	Quality of life scale<br><br>Quality of life scale at BL: 68.9 (16.0)	1 year (1 FU)	Good

Note. Results were obtained directly from the publications, the study's authors or calculated from the data provided in the publications.
AgeCoDe: The German Study on Ageing, Cognition, and Dementia. AUDADIS: Alcohol Use Disorder and Associated Disabilities Interview Schedule. BDI: Beck Depression Inventory. BL: Baseline. CDI: Children's Depression Inventory. CES-D: Center for Epidemiologic Studies Depression Scale. CIDI: Composite International. Diagnostic Interview. CONSTANCES: Cohorte des consultants des Centres d'examens de santé. DASS: Depression, Anxiety and Stress Scales. DIS: Diagnostic Interview Schedule. DRPST: Disaster-Related Psychological Screening Test. DSM: Diagnostic and Statistical Manual of Mental Disorders. EQ-VAS: Visual analogue scale from EuroQuol questionnaire. FDD-DSM-IV: German short version of the Inventory to Diagnose Depression. FU: Follow-up. GAD: generalized anxiety disorder. GDS: Geriatric Depression Scale. GHQ: General Health Questionnaire. HADS: Hospital Anxiety and Depression Scales. HAM-D/HRSD17/HDRS: Hamilton Rating Scale for Depression. ICD: International Statistical Classification of Diseases and Related Health Problems. IES: Impact of Events Scale. LIDO: Longitudinal Investigation of. Depression Outcomes study. MADRS: Montgomery and Asberg Depression Rating Scale. MCS: Mental component summary score. MDAS: Modified Dental Anxiety Scale. MHI-5: Mental Health Inventory. MINI: The Mini-International Neuropsychiatric Interview. MD: Major depression. MOS: Medical Outcomes Study. NEMESIS: The Netherlands Mental Health Survey and Incidence Study. NESARC: The National Epidemiologic Survey on Alcohol and Related Conditions. NOCDA: Netherlands Obsessive Compulsive Disorder Association. PCL: Post-traumatic disorder Checklist. PCS: Physical component summary score. PTSS: Post-traumatic stress symptoms. SASC: Social anxiety Scale for Children. SCL: Symptom Checklist. SF: Short Form Health Survey. PD: panic disorder. PHQ: Patient Health Questionnaire. PREDICT: study on prediction of depression in European general practice attendees. QLDS: Quality of Life in Depression Scale. SAD: social anxiety disorder. SADS: Schedule for Affective Disorders and Schizophrenia. SCAN: Schedules for Clinical Assessment in Neuropsychiatry. SCID: The Structured Clinical Interview. SLAS: Singapore Longitudinal Aging Study. SP: specific phobia. SWAN: Study of Women's Health Across the Nation. WHO: World Health Organization. VETSA: Vietnam Era Twin Study of Aging. WHOQOL: quality of life assessment developed by the World Health Organization Quality of Life Group. Y-BOC: Yale Brown Obsessive Compulsive Scale for Severity.

Appendix. Descriptive table and forest plots for meta-analyses of SF MCS and PCS scores

Figure S1. Forest plot for differences in SF MCS at FU among adults with and without depressive disorders at BL

Study	depression at BL N	Mean	SD	No depression at BL N	Mean	SD	Hedges's g with 95% CI	Weight (%)
Ardal, 2013	14	58	20.1	14	82.5	8.1	-1.55 [-2.38, -0.73]	0.87
Ho, 2014	211	50.5	9.33	1,633	56.1	5.52	-0.92 [-1.07, -0.77]	27.68
Hasche, 2010	266	42.4	11.2	285	52	8.8	-0.96 [-1.13, -0.78]	19.11
Chin, 2015	401	50	12.8	3,104	57.9	7.3	-0.97 [-1.08, -0.87]	52.34
**Overall**							-0.96 [-1.04, -0.88]	

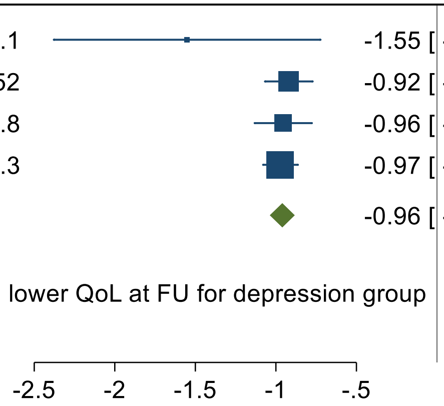

Heterogeneity: $\tau^2 = 0.00$, $I^2 = 0.00\%$, $H^2 = 1.00$

Test of $\theta_i = \theta_j$: Q(3) = 2.30, p = 0.51

Test of $\theta = 0$: z = -24.44, p = 0.00

Random-effects REML model

Figure S2. Forest plot for differences in SF PCS at FU among adults with and without depressive disorders at BL

Study	depression at BL N	Mean	SD	No depression at BL N	Mean	SD	Hedges's g with 95% CI	Weight (%)
Ardal, 2013	14	61.2	17.9	14	76.9	13	-0.97 [-1.74, -0.21]	13.62
Ho, 2014	211	48.4	7.2	1,633	50.9	5.71	-0.42 [-0.57, -0.28]	28.76
Hasche, 2010	266	34.3	9.5	285	38.3	11.3	-0.38 [-0.55, -0.21]	28.33
Chin, 2015	401	41.4	12.9	3,104	50.6	7.8	-1.08 [-1.18, -0.97]	29.29
**Overall**							-0.68 [-1.06, -0.30]	

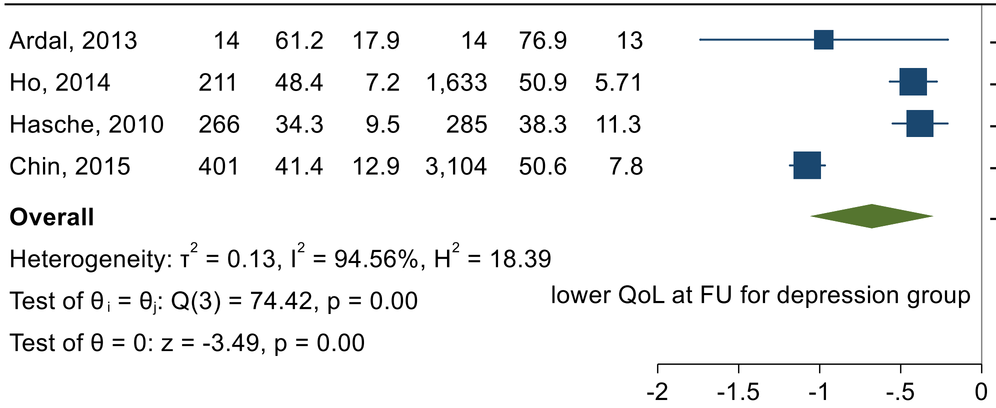

Heterogeneity: $\tau^2 = 0.13$, $I^2 = 94.56\%$, $H^2 = 18.39$

Test of $\theta_i = \theta_j$: Q(3) = 74.42, p = 0.00

Test of $\theta = 0$: z = -3.49, p = 0.00

Random-effects REML model

Appendix. Descriptive table and forest plots for meta-analyses of SF MCS and PCS scores

Figure S3. Forest plot for differences in SF MCS at FU among adults with and without depressive disorders at BL (sensitivity analysis)

	depression at BL			No depression at BL			Hedges's g	Weight
Study	N	Mean	SD	N	Mean	SD	with 95% CI	(%)
Ho, 2014	211	50.5	9.33	1,633	56.1	5.52	-0.92 [-1.07, -0.77]	27.92
Hasche, 2010	266	42.4	11.2	285	52	8.8	-0.96 [-1.13, -0.78]	19.28
Chin, 2015	401	50	12.8	3,104	57.9	7.3	-0.97 [-1.08, -0.87]	52.79
**Overall**							-0.96 [-1.03, -0.88]	

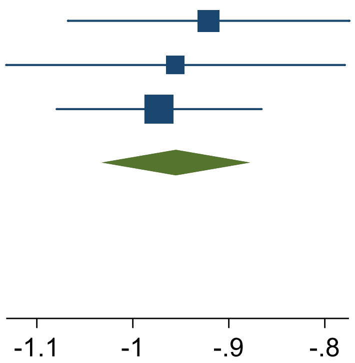

Heterogeneity: $\tau^2 = 0.00$, $I^2 = 0.01\%$, $H^2 = 1.00$

Test of $\theta_i = \theta_j$: Q(2) = 0.31, p = 0.86

Test of $\theta = 0$: z = -24.20, p = 0.00

lower Q

Random-effects REML model

Figure S4. Forest plot for differences in SF PCS at FU among adults with and without depressive disorders at BL (sensitivity analysis)

	depression at BL			No depression at BL			Hedges's g	Weight
Study	N	Mean	SD	N	Mean	SD	with 95% CI	(%)
Ho, 2014	211	48.4	7.2	1,633	50.9	5.71	-0.42 [-0.57, -0.28]	33.30
Hasche, 2010	266	34.3	9.5	285	38.3	11.3	-0.38 [-0.55, -0.21]	32.88
Chin, 2015	401	41.4	12.9	3,104	50.6	7.8	-1.08 [-1.18, -0.97]	33.83
**Overall**							-0.63 [-1.08, -0.19]	

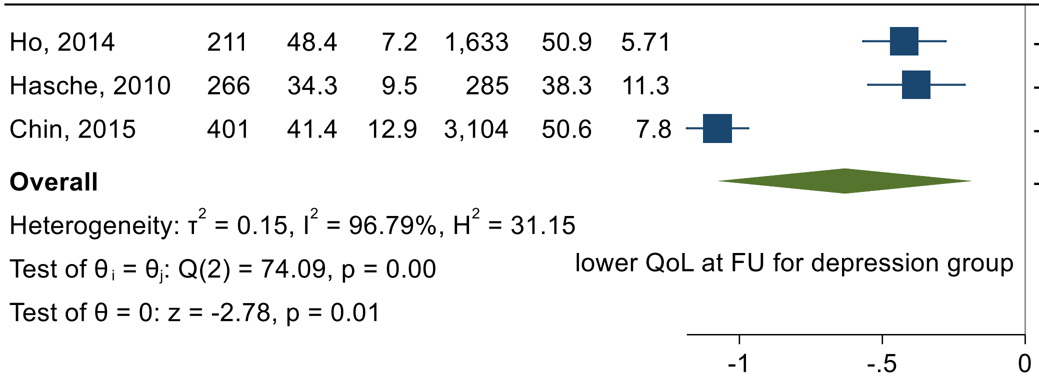

Heterogeneity: $\tau^2 = 0.15$, $I^2 = 96.79\%$, $H^2 = 31.15$

Test of $\theta_i = \theta_j$: Q(2) = 74.09, p = 0.00

Test of $\theta = 0$: z = -2.78, p = 0.01

Random-effects REML model

Appendix. Descriptive table and forest plots for meta-analyses of SF MCS and PCS scores

Figure S5. Forest plot for BL differences in SF MCS among adults with MD at BL with and without remitting courses over time

	Persistent depression			Remission of depression			Hedges's g	Weight
Study	N	Mean	SD	N	Mean	SD	with 95% CI	(%)
De Almeida Fleck, 2005	628	33.4	9.3	340	35	10.6	-0.16 [-0.30, -0.03]	44.26
Hoertel, 2017	944	41.1	12.1	1,643	45	12.16	-0.32 [-0.40, -0.24]	55.74
**Overall**							-0.25 [-0.40, -0.10]	

Heterogeneity: $\tau^2 = 0.01$, $I^2 = 74.95\%$, $H^2 = 3.99$

Test of $\theta_i = \theta_j$: $Q(1) = 3.99$, $p = 0.05$

Test of $\theta = 0$: $z = -3.21$, $p = 0.00$

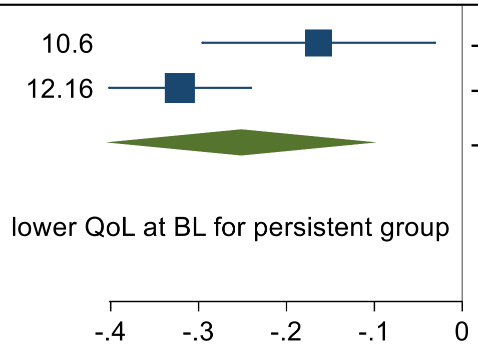

Random-effects REML model

Figure S6. Forest plot for BL differences in SF PCS among adults with MD at BL with and without remitting courses over time

	Persistent depression			Remission of depression			Hedges's g	Weight
Study	N	Mean	SD	N	Mean	SD	with 95% CI	(%)
De Almeida Fleck, 2005	628	40.6	11.1	340	44.2	11.2	-0.32 [-0.46, -0.19]	43.75
Hoertel, 2017	944	46.5	14.1	1,643	48.7	12.16	-0.17 [-0.25, -0.09]	56.25
**Overall**							-0.24 [-0.39, -0.09]	

Heterogeneity: $\tau^2 = 0.01$, $I^2 = 73.14\%$, $H^2 = 3.72$

Test of $\theta_i = \theta_j$: $Q(1) = 3.72$, $p = 0.05$

Test of $\theta = 0$: $z = -3.13$, $p = 0.00$

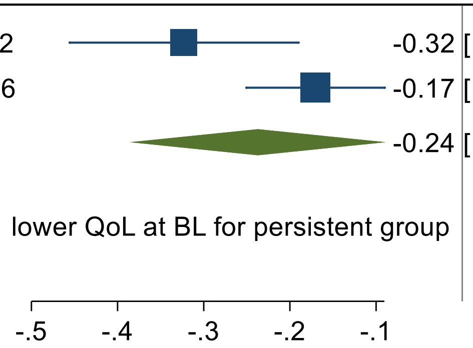

Random-effects REML model

Appendix. Descriptive table and forest plots for meta-analyses of SF MCS and PCS scores

Figure S7. Forest plot for FU differences in SF MCS among adults with depressive disorders at BL with and without remitting courses

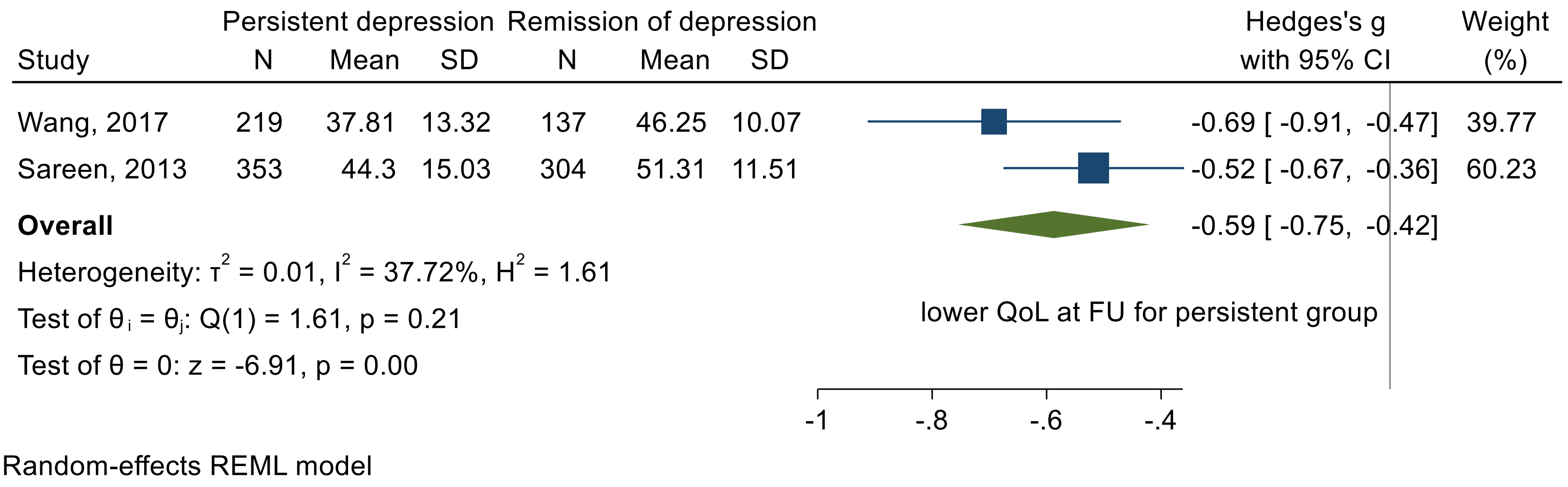

Figure S8. Forest plot for FU differences in SF PCS among adults with depressive disorders at BL with and without remitting courses

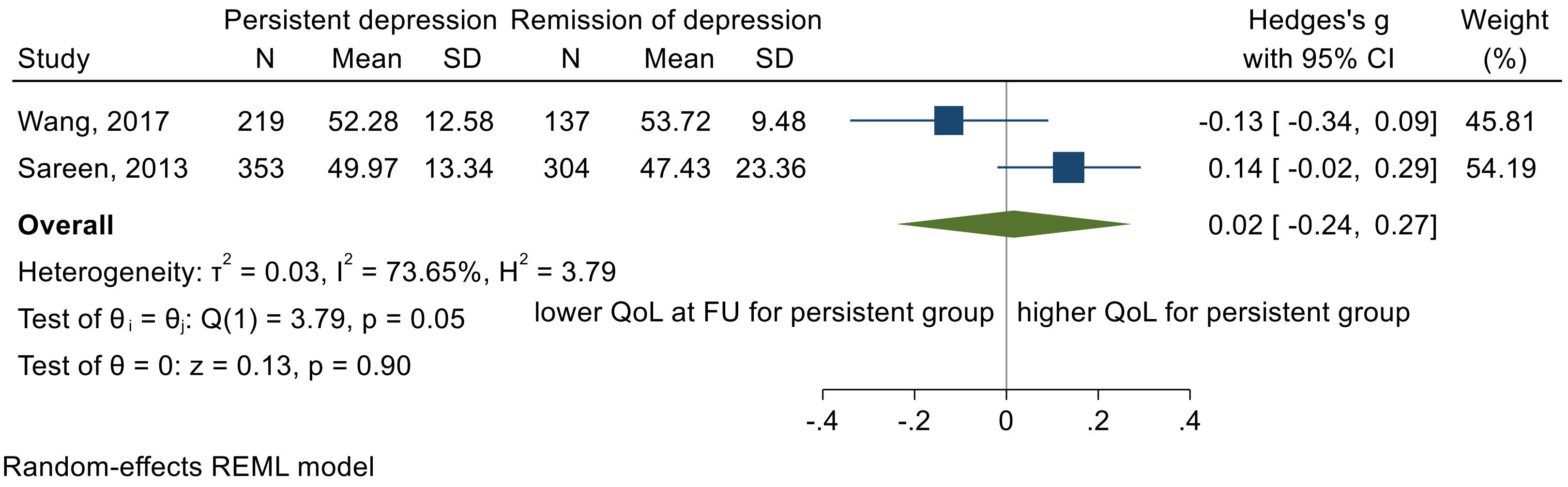

Appendix. Descriptive table and forest plots for meta-analyses of SF MCS and PCS scores

Figure S9. Forest plot for FU differences in SF MCS among adults with anxiety disorders at BL with and without remitting courses

Study	Persistent anxiety N	Mean	SD	Remission of anxiety N	Mean	SD	Hedges's g with 95% CI	Weight (%)
Wang, 2017	312	43.76	16.43	334	51.26	11.88	-0.53 [-0.68, -0.37]	40.22
Sareen, 2013	796	48.4	13.26	1,019	52.6	8.94	-0.38 [-0.47, -0.29]	59.78
**Overall**							-0.44 [-0.58, -0.30]	

Heterogeneity: $\tau^2 = 0.01$, $I^2 = 58.87\%$, $H^2 = 2.43$

Test of $\theta_i = \theta_j$: $Q(1) = 2.43$, $p = 0.12$

Test of $\theta = 0$: $z = -6.16$, $p = 0.00$

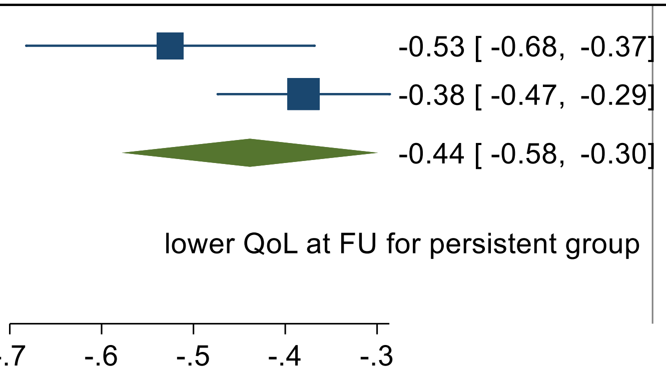

Random-effects REML model

Figure S10. Forest plot for FU differences in SF PCS among adults with anxiety disorders at BL with and without remitting courses

Study	Persistent anxiety N	Mean	SD	Remission of anxiety N	Mean	SD	Hedges's g with 95% CI	Weight (%)
Wang, 2017	312	51.41	13.78	334	52.41	8.77	-0.09 [-0.24, 0.07]	26.55
Sareen, 2013	796	50.11	13.82	1,019	51.29	12.45	-0.09 [-0.18, 0.00]	73.45
**Overall**							-0.09 [-0.17, -0.01]	

Heterogeneity: $\tau^2 = 0.00$, $I^2 = 0.01\%$, $H^2 = 1.00$

Test of $\theta_i = \theta_j$: $Q(1) = 0.00$, $p = 0.97$

Test of $\theta = 0$: $z = -2.21$, $p = 0.03$

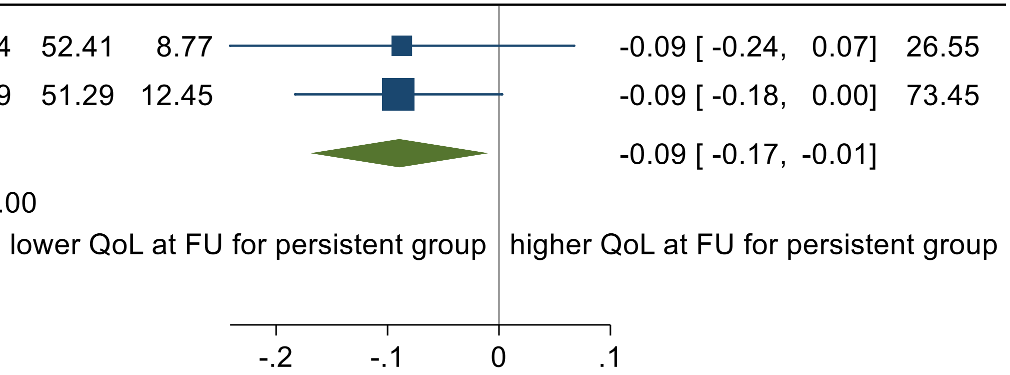

Random-effects REML model
